# Periprocedural Outcomes Associated With Use of a Left Atrial Appendage Occlusion Device in China

**DOI:** 10.1001/jamanetworkopen.2022.14594

**Published:** 2022-05-31

**Authors:** Fangju Su, Chao Gao, Jianzheng Liu, Zhongping Ning, Beng He, Yi Liu, Yawei Xu, Bing Yang, Yuechun Li, Junfeng Zhang, Xianxian Zhao, Yushun Zhang, Hao Hu, Xianfeng Du, Ruiqin Xie, Ling Zhou, Jie Zeng, Zhongbao Ruan, Haitao Liu, Jun Guo, Rutao Wang, Scot Garg, Osama Soliman, David R. Holmes, Patrick W. Serruys, Ling Tao

**Affiliations:** 1 Department of Cardiology, Xijing Hospital, Xi’an, China; 2 Department of Cardiology, The 940th Hospital, Lanzhou, China; 3 Department of Cardiology, Shanghai University of Medicine & Health Sciences Affiliated Zhoupu Hospital, Shanghai, China; 4 Department of Cardiology, Shanghai Chest Hospital, Shanghai Jiao Tong University, Shanghai, China; 5 Department of Cardiology, Shanghai Tenth People’s Hospital, Shanghai Jiaotong University School of Medicine, Shanghai, China; 6 Department of Cardiology, Shanghai East Hospital, School of Medicine, Tongji University, Shanghai, China; 7 Department of Cardiology, Second Affiliated Hospital and Yuying Children’s Hospital of Wenzhou Medical University, Wenzhou, China; 8 Department of Cardiology, Shanghai Ninth People’s Hospital, Shanghai Jiaotong University School of Medicine, Shanghai, China; 9 Department of Cardiology, Changhai Hospital, Navy Military Medical University, Shanghai, China; 10 Department of Cardiology, The First Affiliated Hospital of Xi’an Jiaotong University, Xi’an, China; 11 Department of Cardiology, The Second Affiliated Hospital of Lanzhou University, Lanzhou, China; 12 Department of Cardiology, Arrhythmia Center, Ningbo First Hospital, Ningbo, China; 13 Department of Cardiology, The Second Affiliated Hospital of Hebei Medical University, Shijiazhuang, China; 14 Department of Cardiology, Nanjing First Hospital, Nanjing, China; 15 Department of Cardiology, Sichuan Provincial People's Hospital, University of Electronic Science and Technology of China, Chengdu, China; 16 Department of Cardiology, Taizhou People’s Hospital, Taizhou, China; 17 Department of Cardiology, Senior Department of Cardiology, the Sixth Medical Center of PLA General Hospital, Beijing, China; 18 Department of Cardiology, Royal Blackburn Hospital, Blackburn, United Kingdom; 19 Department of Cardiovascular Diseases and Internal Medicine, Mayo Clinic, Rochester, Minnesota; 20 Department of Cardiology, National University of Ireland, Galway, Ireland

## Abstract

**Importance:**

Left atrial appendage occlusion (LAAO) has emerged as an alternative to anticoagulation for patients with atrial fibrillation. However, the performance of LAAO among East Asian patients is unknown.

**Objective:**

To document the procedural success rate and configurations, major adverse event rates, and antithrombotic medication regimens during and after LAAO procedures among patients in China.

**Design, Setting, and Participants:**

In this cohort study, consecutive patients from 39 Chinese centers undergoing LAAO were prospectively enrolled between April 1, 2019, and October 31, 2020. Periprocedural and intraprocedural techniques and postprocedural medications were left to the surgeon’s discretion. Data were analyzed from July 1 to November 1, 2021.

**Exposure:**

LAAO for patients with atrial fibrillation.

**Main Outcomes and Measures:**

The main outcomes were procedural success and complication rates periprocedure and major adverse event rates of death, stroke, systemic embolism, and bleeding events at 30 days postprocedure; the composite end point of death, stroke, and systemic embolism was also analyzed. Unadjusted and multivariable-adjusted logistic regression analyses were performed to assess the associations of periprocedural techniques (types of anesthesia, intraprocedural imaging modalities, and combined ablation procedure) with 30-day adverse events.

**Results:**

Among 3096 enrolled participants, 1782 participants (57.6%) were men, and the mean (SD) age was 69 (9) years. Participants had a high risk of stroke (mean [SD] cardiovascular risk score, 4.0 [1.8]) and a moderate-to-high risk of bleeding (mean [SD] bleeding risk score, 2.4 [1.2]). A total of 1287 procedures (41.6%) were performed under local anesthesia, while 493 procedures (15.9%) used only fluoroscopy guidance. In 1297 procedures (41.9%), LAAO implantation was combined with radiofrequency ablation or cryoablation for atrial fibrillation. Procedural success was achieved in 3032 patients (97.9%). At 30-day follow-up, the rate of the composite end point of death, stroke, or systemic embolism was 0.52% (95% CI, 0.32%-0.84%), and the rate of any life-threatening or major bleeding was 1.23% (95% CI, 0.90%-1.68%). No significant associations were observed between the procedural success or 30-day adverse events and the types of anesthesia (general or local), intraprocedural imaging (transesophageal echocardiography, fluoroscopy, or intracardiac echocardiography), or whether a combined ablation procedure was performed or not. In centers performing at least 40 procedures per year, compared with those performing fewer than 40 procedures per year, procedural success was significantly higher (adjusted odd ratio [aOR], 1.97; 95% CI, 1.01-3.53; *P* = .02) and risk of life-threatening or major bleeding was significantly lower (aOR, 0.42; 95% CI, 0.21-0.87; *P* = .02).

**Conclusions and Relevance:**

These findings suggest that patients with a high risk of stroke and moderate to high risk of bleeding who underwent implantation of a LAAO device in Chinese centers had high rates of procedural success and low rates of short-term ischemic and bleeding events.

## Key Points

**Question:**

What are clinical outcomes and procedural success rates associated with percutaneous left atrial appendage occlusion (LAAO) among East Asian individuals?

**Findings:**

In this cohort study of 3096 consecutively enrolled participants in China, LAAO was associated with a high procedural success rate and a low 30-day adverse event rate. Intraprocedural variations with the type of anesthesia, modality of image guidance, or whether a combined ablation procedure was performed or not had no associations with procedural success or thrombotic and bleeding events at 30 days.

**Meaning:**

These findings suggest that LAAO among patients in China had a high procedural success rate and low major adverse event rates regardless of procedural configurations.

## Teaser

This cohort study assesses the procedural success and major adverse event rates associated with left atrial appendage occlusion procedures among patients in China.

## Introduction

Oral anticoagulant (OAC) medication is the standard of care in patients with nonvalvular atrial fibrillation (AF) who are at risk of stroke; however, there are multiple groups of patients in whom an alternative strategy is required. This need arises for several reasons, including contraindications to OAC, adverse effects from OAC, adherence, and quality-of-life issues.^1^

Percutaneous left atrial appendage occlusion (LAAO) is a nonpharmacological strategy for stroke prevention in patients with AF, and data from randomized trials and meta-analyses have suggested that LAAO has comparable efficacy to warfarin^2,3,4,5^ and novel OACs (NOACs).^6,7^ Data from several registries have also confirmed the safety of LAAO, with low rates of complications.^5,8,9,10,11,12^ Current European Society of Cardiology (ESC)^13^ and American College of Cardiology (ACC)^14^ guidelines recommend LAAO for patients with nonvalvular AF who have contraindications or are unsuitable for long-term OAC (class IIb recommendations).

East Asian individuals are one of the largest ethnic groups, with a population in excess of 1.5 billion.^15^ Compared with other ethnic groups, East Asian individuals have a unique risk-benefit trade-off in the management of stroke prevention in nonvalvular AF, with reduced anti-ischemic benefits and increased bleeding risk with antithrombotic therapies, particularly intracranial bleeding, known as the “East Asian paradox”^15^; the adherence with OAC is also commonly suboptimal.^16,17^ Consequently, East Asian patients with nonvalvular AF may have a greater benefit from nonpharmacological strategies for stroke prevention, such as implantation of an LAAO device.^18^ However, clinical outcomes from LAAO implants in East Asian, Chinese, Korean, Japanese, or other ethnic groups have been less well-documented in large cohorts.^18,19,20,21^ How applicable the results from prior randomized clinical trials^2,4^ and registries^5,8,9,10,11,12,22^ are to East Asian patients is questionable, given that these studies primarily enrolled White populations (between 92% to 94%).

In 2013, WATCHMAN (Boston Scientific) became the first approved LAAO device in China, and by 2017, approximately 2000 implant procedures were being performed annually.^23^ The Registry to Evaluate Chinese Real-World Clinical Outcomes in Patients With AF Using the WATCHMAN Left Atrial Appendage Closure Technology (RECORD) study was designed to prospectively include approximately 3000 consecutive patients from 39 Chinese centers, with the aim to examine the safety and efficacy of the WATCHMAN LAAO device in the Chinese population. The follow-up is ongoing, and we plan to continue for up to 5 years. In this study, the periprocedural and 30-day clinical outcomes and their associations with different types of periprocedural techniques are reported.

## Methods

This cohort study adhered to the international rules for scientific studies and the Declaration of Helsinki.^24^ Central or local ethics committee approval was obtained in all participating centers. All participants provided informed consent prior to the procedure. This study is reported following the Strengthening the Reporting of Observational Studies in Epidemiology (STROBE) reporting guideline for cohort studies.

### Study Population

The RECORD study (NCT03917563) was a multicenter, prospective, nonrandomized cohort study that included 3096 patients from 39 centers in China from April 1, 2019, to October 31, 2020. Consecutive patients were recruited from each participating center, with patients suitable for inclusion if they were eligible to receive the WATCHMAN device per their physician’s discretion, in accordance with appropriate local and international guidelines, and were of legal age to provide informed consent. Periprocedural and intraprocedural techniques and postprocedural medications were left to the surgeon’s discretion. A total of 159 surgeons with varying levels of experience implanting the device participated in the study.

### Outcomes

The objective was to obtain data on device, technical, and procedural success; periprocedural complications; and outcomes at 30 days after the procedure. All assessed events, including death, stroke, systemic embolism, transient ischemic attacks (TIA), procedural complications, and bleeding events, were adjudicated by an independent clinical event committee of 5 physicians with expertise in electrophysiology and/or interventional cardiology. The adjudication of events was based on the definitions included in the consensus document of percutaneous LAAO: the Munich consensus document^25^ on definitions, end points, and data collection requirements for clinical studies. Bleeding was evaluated by both the Munich consensus document^25^ and the Bleeding Academic Research Consortium criteria.^26^ Device success was defined as the device deployed and implanted in the correct position. Technical success was defined as the exclusion of the left atrial appendage, with no device-related complications and no leak greater than 5 mm (as reported by each site). Procedural success was defined as technical success and no procedure-related complications. The general anesthesia group included patients who had mechanical ventilation; patients with monitored anesthesia care or sedation without mechanical ventilation were included in the local anesthesia group.

### Statistical Analysis

Continuous variables with normal distribution are expressed as mean and SD or described as median and IQR. Categorical variables are presented as counts and percentages and are compared by Fisher exact test when appropriate. To investigate the association of intraprocedural configurations with outcomes, univariable and multivariable logistic regression were used; variables included in the multivariable model 1 were HAS-BLED score (calculated as hypertension, abnormal kidney or liver function, stroke, bleeding history or predisposition, labile international normalized ratio, elderly [ie, aged >65 years], and using drugs or alcohol concomitantly) and CHA_2_DS_2_-VASc score (calculated as congestive heart failure, hypertension, age ≥75 years, diabetes, stroke or TIA, vascular disease, age 65-74 years, and sex category). Variables included in model 2 were those representing demographic characteristics (age, sex), coexisting medical conditions (diabetes, hypertension, prior coronary artery disease, prior vascular disease, previous stroke, chronic heart failure), type of anesthesia, type of intraprocedural imaging guidance, combined procedure, the center volume, HAS-BLED score, and CHA_2_DS_2_-VASc score. No missing data were imputed.

Analyses were performed using SAS statistical software version 9.4 (SAS Institute) and R statistical software version 4.1 (R Project for Statistical Computing). A 2-sided *P* < .05 was considered statistically significant. Data were analyzed from July 1 to November 1, 2021.

## Results

### Baseline Characteristics

From April 1, 2019, until October 31, 2020, 3569 consecutive patients with an indication for an LAAO device were screened for enrollment in the 39 participating centers. Of these, 168 patients refused to provide consent, 162 patients declined to adhere to follow-up owing to the COVID-19 pandemic, and 143 patients were participating in other studies; these patients were excluded, leaving a total of 3096 participants in the study, with 30-day follow-up completed in 3062 participants (98.9%) (eFigure 1 in the Supplement).

Baseline demographics and procedural characteristics are summarized in Table 1. The mean (SD) age of participants was 69 (9) years, and 1782 participants (57.6%) were men. Participants were at high risk of stroke, as reflected by relatively high rates of hypertension (2130 participants [68.8%]), vascular disease (1681 participants [54.5%]), previous ischemic stroke or TIA (1380 participants [44.7%]), and diabetes (720 participants [23.3%]), and a mean (SD) CHA_2_DS_2_-VASc score of 4.0 (1.8) (eFigure 2 in the Supplement). Participants were also at a moderate to high risk of bleeding, with 1308 participants (42.3%) having had a previous ischemic or hemorrhagic stroke and 314 participants (10.2%) had prior bleeding or predisposition. The mean (SD) HAS-BLED score was 2.4 (1.2), with 1397 participants (45.2%) having a HAS-BLED score of 3 or greater (eFigure 2 in the Supplement).

**Table 1.** Baseline and Procedural Characteristics

Characteristic	Patients, No./total No. (%)
Age, y^a^	
Mean (SD)	69.1 (9.4)
65-74	1313/3096 (42.4)
≥75	914/3096 (29.5)
Gender	
Women	1314/3096 (42.4)
Men	1782/3096 (57.6)
Body mass index, mean (SD)^a^	24.8 (3.5)
Heart rate, mean (SD), beats per min^a^	82.0 (20.5)
Diabetes	720/3096 (23.3)
Previous stroke^a^	
Any	1418/3090 (45.9)
Ischemic stroke or TIA	1380/3090 (44.7)
Hemorrhagic stroke	107/3090 (3.5)
Hypertension	2130/3094 (68.8)
Coronary artery disease	879/3086 (28.5)
Previous PCI	327/3086 (10.6)
Previous CABG	46/3086 (1.5)
Vascular disease^b^	1681/3086 (54.5)
Current smoker	334/3094 (10.8)
Alcohol abuse	169/3092 (5.5)
Chronic heart failure	462/3089 (15.0)
LVEF, mean (SD), %^a^	60.0 (8.3)
Abnormal thyroidal function	133/3068 (4.3)
Abnormal kidney function	72/3077 (2.3)
Abnormal liver function	51/3085 (1.7)
Bleeding history or predisposition^c^	314/3093 (10.2)
Concomitant use of drugs	1041/3061 (34.0)
Classification of AF	
Paroxysmal	1249/3096 (40.3)
Persistent	1277/3096 (41.2)
Long-standing persistent (>1 y) or permanent	570/3096 (18.4)
CHA_2_DS_2_-VASc score, mean (SD)^a^	4.0 (1.8)
HAS-BLED score, mean (SD)^a^	2.4 (1.2)
ATRIA score, mean (SD)^a^	6.2 (2.9)
Recaptured before release (≥2 times)	258/3096 (8.3)
Device used^d^	3205/3096
Kissing devices	5/3082 (0.1)
Device size, mm	
21	188/3082 (6.1)
24	634/3082 (20.6)
27	918/3082 (29.8)
30	714/3082 (23.2)
33	633/3082 (20.5)
Residual peridevice leakage	
Complete sealing^e^	2770/3082 (89.9)
Leak, <3mm	249/3082 (8.1)
Leak, 3-5 mm	60/3082 (1.9)
Leak, >5 mm	3/3082 (0.1)
Anesthesia	
General	1809/3096 (58.4)
Localized	1287/3096 (41.6)
Preprocedural screening	
CTA	1812/3096 (58.5)
TEE	1756/3096 (56.7)
None	0/3096
Imaging guidance	
TEE	2508/3096 (81.0)
Fluoroscopy	493/3096 (15.9)
ICE	95/3096 (3.1)
Combined procedures (1-staged)	
Radiofrequency ablation or cryoablation	1297/3096 (41.9)
ASD or PFO occlusion	78/3096 (2.5)
PCI or PTCA	51/3096 (1.7)
Pacemaker implantation	8/3096 (0.3)
Percutaneous mitral valvuloplasty	5/3096 (0.2)
TAVR	2/3096 (0.1)
Others^f^	6/3096 (0.2)
Total	1450/3096 (46.8)

Abbreviations: AF, atrial fibrillation; ASD, atrial septal defect; ATRIA, Anticoagulation and Risk Factors in Atrial Fibrillation; CABG, coronary artery bypass graft; CHA_2_DS_2_-VASc, congestive heart failure, hypertension, age 75 years or older, diabetes, stroke or transient ischemic attack, vascular disease, age 65 to 74 years, sex category; CTA, computed tomography angiography; HAS-BLED, hypertension, abnormal kidney or liver function, stroke, bleeding history or predisposition, labile international normalized ratio, elderly (ie, aged >65 years), and using drugs or alcohol concomitantly; ICE, intracardiac echocardiography; LVEF, left ventricular ejection fraction; PCI, percutaneous coronary intervention; PFO, patent foramen ovale; PTCA, percutaneous transluminal coronary angioplasty; TAVR, transcatheter aortic valve replacement; TEE, transesophageal echocardiography; TIA, transient ischemic attack.

^a^ Among 3096 patients, except for BMI (2789 patients), LVEF (2786 patients), CHA_2_DS_2_ and CHA_2_DS_2_-VASc scores (3065 patients), and HAS-BLED and ATRIA scores (3068 patients).

^b^ Vascular disease includes previous myocardial infarction, peripheral artery disease, or aortic plaque, defined according to the CHA_2_DS_2_-VASc score.

^c^ Bleeding history or predisposition includes previous major hemorrhage or anemia or severe thrombocytopenia, defined according to the HAS-BLED score.

^d^ Mean of 1.04 per patient.

^e^ Site reported according to immediate postprocedure evaluation by TEE (2495 patients), ICE (95 patients), or fluoroscopy (492 patients).

^f^ Others included femoral artery stent implantation (1 patient), implantation of vena cava filter (1 patient), splenic artery angiography (1 patient), renal angiography (1 patient), radiofrequency ablation of supraventricular tachycardia (1 patient), electrocardiogram event recorder implantation (1 patient).

A total of 1812 participants (58.5%) had preprocedural computed tomography angiography screening, while 1756 participants (56.7%) had transesophageal echocardiography (TEE) screening. Sites reported complete sealing of the LAA in 2770 of 3082 procedures (89.9%). Procedures were performed under local anesthesia in 1287 participants (41.6%), while intraprocedure image guidance was performed using TEE in 2508 participants (81.0%), fluoroscopy alone in 493 participants (15.9%), and intracardiac echo (ICE) in 95 participants (3.1%) (eFigure 3 in the Supplement). In 1450 participants (46.8%), the LAAO device implantation was combined with another procedure, which was most commonly an AF ablation (1297 participants [41.9%]).

### Post-LAAO Medication

Discharge medication regimens were heterogenous, with the commonest being NOAC monotherapy, followed by a NOAC plus aspirin or a P2Y12 inhibitor, OAC monotherapy, dual antiplatelet therapy (DAPT, and finally OAC plus aspirin or a P2Y12 inhibitor (Figure, A). Details of medication after LAAO are presented in eTable 1 and eTable 2 in the Supplement. Postprocedural adherence rates to antithrombotic medication according to the ESC guidelines for the diagnosis and management of AF^13^ were 6.2% and the Chinese Society of Cardiology (CSC) expert consensus on LAA closure (eTable 3 in the Supplement)^27^ were 39.0% (Figure, B). Participants with a HAS-BLED score of at least 3 who were adherent with post-LAAO medication recommendations from the CSC expert consensus had a lower rate of death, stroke, systemic embolism, and any life-threatening or major bleeding, compared with those who were nonadherent (adjusted odds ratio [aOR], 2.21; 95% CI, 1.02-4.77; *P* = .045) at 30 days (eTable 4 and eTable 5 in the Supplement).

**Figure.**
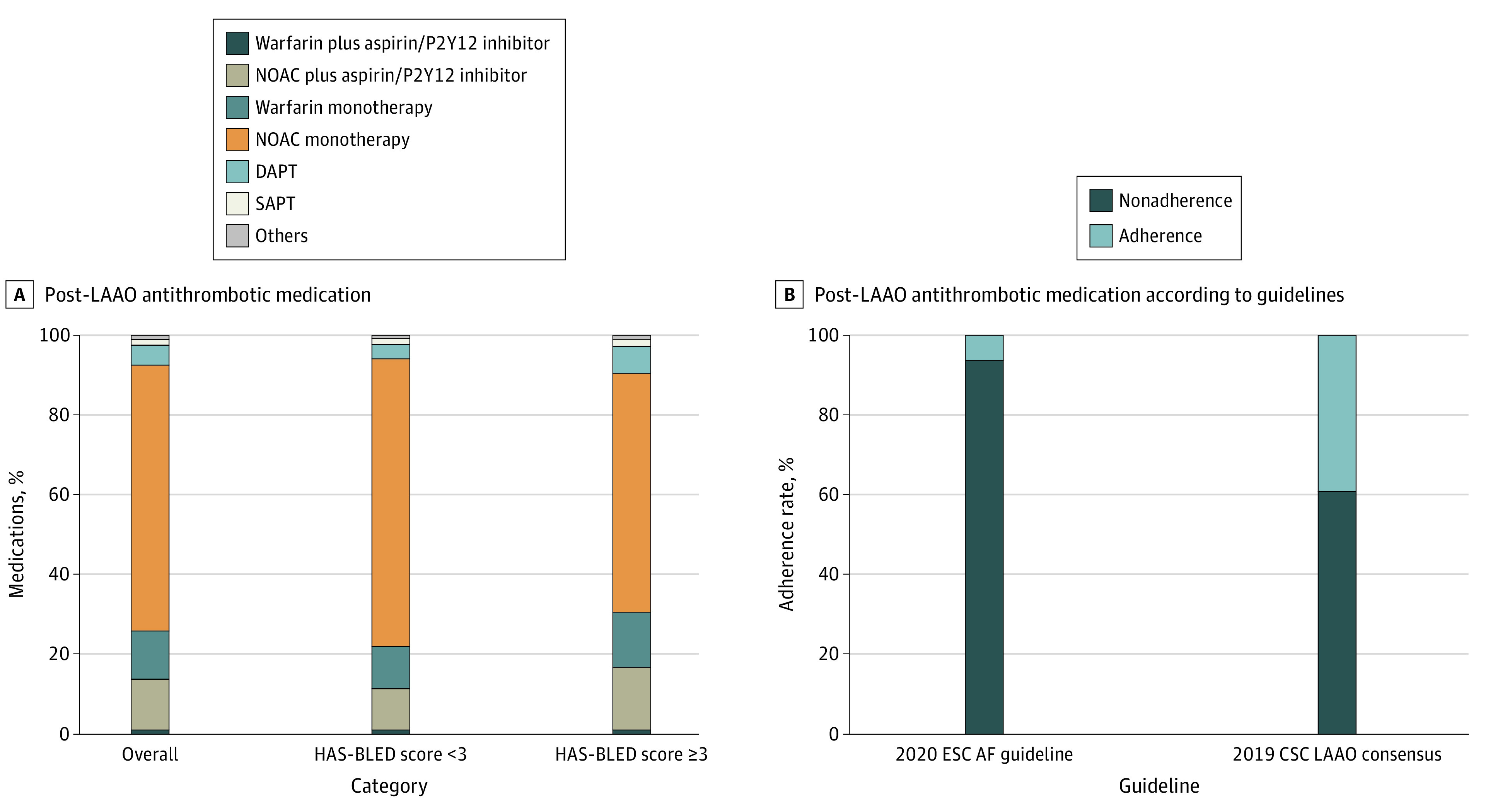
Post–Left Atrial Appendage Occlusion (LAAO) Antithrombotic Medications AF indicates atrial fibrillation; CSC, Chinese Society of Cardiology^27^; DAPT, dual antiplatelet therapy; ESC, European Society of Cardiologists^13^; HAS-BLED, hypertension, abnormal kidney or liver function, stroke, bleeding history or predisposition, labile international normalized ratio, elderly (ie, aged >65 years), and using drugs or alcohol concomitantly; NOAC, novel oral anticoagulant; and SAPT, single antiplatelet therapy.

### Implantation Success and Short-term Clinical Outcomes

Implant success rates are shown in Table 2 and eFigure 4 in the Supplement. Device success was achieved in 3082 participants (99.5%; 95% CI, 99.3%-99.8%), with 14 participants (0.4%) experiencing deployment failures owing to unfavorable anatomy or a mismatch between the size of the device and the LAA. Technical success was accomplished in 3067 participants (99.1%; 95% CI, 98.7%-99.3%); 12 participants had device-related complications, and 3 participants residual flow greater than 5 mm. Procedural success was reached in 3032 participants (97.9%; 95% CI, 97.4%-98.4%), with 36 participants having procedure-related complications.

**Table 2.** Clinical Events at 30 Days After LAOO Procedure

Outcome	Events		Total	
	≤7 d	8-30 d	Events	Event rate (95% CI)
Device success	NA	NA	3082	99.5 (99.3-99.8)
Technical success	NA	NA	3068	99.1 (98.8-99.4)
Procedure success	NA	NA	3032	97.9 (97.4-98.4)
Death, stroke, or systemic embolism	9	7	16	0.52 (0.30-0.84)
Death, stroke, or systemic embolism and any life-threatening or major bleeding	40	10	50	1.61 (1.20-2.12)
Death				
All-cause	2	6	8	0.26 (0.11-0.51)
Cardiovascular death				
Any	2	5	7	0.23 (0.09-0.47)
Pulmonary embolism	0	1	1	NA
Cardiac arrest	2	1	3	NA
Hemorrhagic stroke	0	1	1	NA
Gastrointestinal bleeding	0	1	1	NA
Unknown cause	0	1	1	NA
Noncardiovascular death^a^	0	1	1	0.03 (0.00-0.18)
Stroke				
Any	7	2	9	0.29 (0.13-0.55)
Hemorrhagic stroke	2	1	3	0.10 (0.02-0.28)
Ischemic stroke	5	1	6	0.19 (0.07-0.42)
TIA	1	1	2	0.06 (0.01-0.23)
Systemic embolism	0	1	1	0.03 (0.00-0.18)
Procedural complications				
Any	37	0	37	1.20 (0.84-1.64)
Vascular access-related	3	0	3	0.10 (0.02-0.28)
Device-related	12	0	12	0.39 (0.20-0.68)
Cardiac tamponade	11	0	11	NA
Pneumothorax	1	0	1	NA
Pericardial effusion	20	0	20	0.65 (0.40-1.00)
Others	2	0	2	0.06 (0.01-0.23)
Esophageal	1	0	1	NA
Adverse reaction to anesthesia	1	0	1	NA
Readmission	15	48	63	2.03 (1.57-2.60)
Any bleeding	39	13	52	1.68 (1.26-2.20)
LAAO Munich consensus classification				
Any life-threatening or major bleeding	35	4	38	1.23 (0.87-1.68)
Life threatening or disabling				
Any	2	3	5	0.16 (0.05-0.38)
Intracranial	2	1	3	NA
Intraocular	0	1	1	NA
Gastrointestinal	0	1	1	NA
Major bleeding	33	0	33	1.06 (0.73-1.49)
Pericardial bleeding				
With tamponade	11	0	11	NA
Without tamponade	20	0	20	NA
Femoral artery	2	0	2	NA
Minor bleeding	3	11	14	0.45 (0.25-0.76)
BARC classification				
Type 5	0	2	2	0.06 (0.01-0.23)
Type 3	15	1	16	0.52 (0.30-0.84)
Type 3c	2	1	3	NA
Type 3b	13	0	13	NA
Type 2	24	10	34	1.10 (0.76-1.53)

Abbreviations: BARC, Bleeding Academic Research Consortium; NA, not applicable; LAAO, left atrial appendage occlusion; TIA, transient ischemic attacks.

^a^ One noncardiovascular death was pneumonia.

Death, stroke, or a systemic embolism occurred in 9 patients in the first 7 days after their procedure (Table 2), including 2 cardiovascular deaths caused by cardiac arrest, 2 hemorrhagic strokes, and 5 ischemic strokes. There were 2 life-threatening or disabling bleeding events and 33 major bleeding events. A total of 37 patients experienced procedural complications, including 3 related to vascular access, 12 device-related complications, and 20 pericardial effusions. Between 8 and 30 days, there were 6 additional deaths, including 1 death from hemorrhagic stroke and 1 death from ischemic stroke. At the 30-day follow-up, the all-cause mortality rate was 0.26% (95% CI, 0.11-0.51), the rate of a composite end point of death, stroke, and systemic embolism was 0.52% (95% CI, 0.30-0.84), and the rate of any life-threatening or major bleeding was 1.23% (95% CI, 0.87-1.68).

### Center Experiences and Procedural Configurations

The median (IQR) annual number of LAAO procedures performed per center was 40 (16-80) procedures. eTable 6 and eTable 7 in the Supplement showed a comparison among baseline characteristics when centers were divided into 5 strata according to annual volume (<20, 20-39, 40-59, 60-79, and ≥80 procedures per year), and eFigure 5 in the Supplement shows rates of procedural success according to center volume. Procedural success was significantly higher (aOR, 1.97; 95% CI, 1.10-3.53; *P* = .02) and life-threatening or major bleeding was significantly lower (aOR, 0.42; 95% CI, 0.21-0.87; *P* = .02) in centers performing 40 or more procedures annually vs those performing fewer than 40 procedures annually. (Table 3) Analyses according to surgeon experience are shown in eFigure 6 in the Supplement.

**Table 3.** Associations of Procedural Configurations With Outcomes

Procedural configuration	Events/No. (%)	Univariable		Multivariable model 1^a^		Multivariable model 2^b^		
		OR (95% CI)	*P* value	OR (95% CI)	*P* value	OR (95% CI)	*P* value
**Procedural success**									
Anesthesia							
General	1770/1809 (97.8)	1 [Reference]	.68	1 [Reference]	.72	1 [Reference]	.73
Local	1262/1287 (98.1)	1.11 (0.67-1.85)		0.90 (0.50-1.61)		0.90 (0.50-1.62)	
Periprocedural imaging modality							
Fluoroscopy	487/493 (98.8)	1 [Reference]	NA	1 [Reference]	NA	1 [Reference]	NA
TEE	2452/2508 (97.8)	0.54 (0.23-1.26)	.15	0.55 (0.21-1.43)	.22	0.57 (0.22-1.49)	.25
ICE	93/95 (97.9)	0.57 (0.11-2.88)	.50	0.75 (0.14-3.90)	.73	0.87 (0.17-4.58)	.87
Combined procedure							
No	1763/1799 (98.0)	1 [Reference]	.76	1 [Reference]	.72	1 [Reference]	.48
Ablation procedure combined^c^	1269/1297 (97.8)	0.93 (0.56-1.52)		0.91 (0.54-1.52)		0.83 (0.49-1.40)	
Center volume, LAAO procedures/y^d^							
<40	494/512 (96.5)	1 [Reference]	.01	1 [Reference]		1 [Reference]	.02
≥40	2538/2584 (98.2)	2.01 (1.16-3.50)		1.94 (1.09-3.45)	.02	1.97 (1.10-3.53)	
**Death, stroke, or systemic embolism at 30 d**									
Anesthesia							
General	10/1809 (0.6)	1 [Reference]	.74	1 [Reference]	.91	1 [Reference]	.67
Local	6/1287 (0.5)	0.84 (0.31-2.32)		1.07 (0.33-3.46)		1.31 (0.39-4.47)	
Periprocedural imaging modality							
Fluoroscopy	2/493 (0.4)	1 [Reference]	NA	1 [Reference]	NA	1 [Reference]	NA
TEE	14/2508 (0.6)	1.38 (0.31-6.08)	.67	1.47 (0.26-8.33)	.663	1.49 (0.25-8.77)	.66
ICE	0/95	NA	.98	NA	.98	NA	.98
Combined procedure							
No	8/1799 (0.4)	1 [Reference]	.51	1 [Reference]	.40	1 [Reference]	.30
Ablation procedure combined^c^	8/1297 (0.6)	1.39 (0.52-3.71)		1.54 (0.56-4.25)		1.77 (0.60-5.18)	
Center volume, LAAO procedures/y^d^							
<40	3/512 (0.6)	1 [Reference]	.81	1 [Reference]	.79	1 [Reference]	.94
≥40	13/2584 (0.5)	0.86 (0.24-3.02)		0.84 (0.23-3.06)		0.95 (0.25-3.58)	
**Any life-threatening or major bleeding at 30 d**									
Anesthesia							
General	22/1809 (1.2)	1 [Reference]	.95	1 [Reference]	.56	1 [Reference]	.52
Local	16/1287 (1.2)	1.02 (0.53-1.96)		1.25 (0.60-2.60)		1.28 (0.61-2.69)	
Periprocedural imaging modality							
Fluoroscopy	3/493 (0.6)	1 [Reference]	NA	1 [Reference]	NA	1 [Reference]	NA
TEE	33/2508 (1.3)	2.18 (0.67-7.13)	.20	2.15 (0.59-7.88)	.25	2.25 (0.61-8.31)	.22
ICE	2/95 (2.1)	3.51 (0.58-21.31)	.17	2.32 (0.36-14.76)	.37	2.03 (0.31-13.28)	.46
Combined procedure							
No	21/1646 (1.2)	1 [Reference]	.98	1 [Reference]	.84	1 [Reference]	.85
Ablation procedure combined^c^	16/1296 (1.2)	1.01 (0.53-1.93)		0.96 (0.49-1.87)		1.07 (0.54-2.10)	
Center volume, LAAO procedures/y^d^							
<40	13/512 (2.5)	1 [Reference]	.005	1 [Reference]	.01	1 [Reference]	.02
≥40	25/2584 (1.0)	0.38 (0.19-0.74)		0.41 (0.20-0.82)		0.42 (0.21-0.87)	

Abbreviations: ICE, intracardiac echocardiography; LAAO, left atrial appendage occlusion; NA, not applicable; TEE, transesophageal echocardiography.

^a^ Model 1 covariates were the type of anesthesia, type of guidance, combined procedure, the volume of centers, HAS-BLED (hypertension, abnormal kidney or liver function, stroke, bleeding history or predisposition, labile international normalized ratio, elderly (ie, aged >65 years), and using drugs or alcohol concomitantly) score, and CHA_2_DS_2_-VASc (congestive heart failure, hypertension, age ≥75 years, diabetes, stroke or transient ischemic attack, vascular disease, age 65-74 years, sex category) score.

^b^ Model 2 covariates were the type of anesthesia, type of guidance, combined procedure, the volume of centers, age, sex, diabetes, coronary artery disease, vascular disease, previous stroke, hypertension, heart failure, CHA_2_DS_2_-VASc score, and HAS-BLED score.

^c^ Ablation procedure combined included radiofrequency ablation or cryoablation.

^d^ Strata was defined according to the median of LAAO number performed during the RECORD study.

The association of periprocedural and intraprocedural configurations with 30-day outcomes are shown in Table 3. Rates of procedural success; the composite end point of death, stroke, and systemic embolism; and any life-threatening or major bleeding did not differ by general vs local anesthesia, imaging guidance type, or a single or combination procedure. The results of exploratory subgroup analyses are shown in eTables 8 through 13 in the Supplement.

## Discussion

This cohort study found that there was a high procedural success rate of 97.9% for implanting the WATCHMAN percutaneous LAAO device in this large population of patients in China. On discharge, most patients (66%) were receiving NOAC monotherapy, with adherence rates with postoperative antithrombotic medications according to the ESC AF guidelines^13^ at 6.2% and with the CSC expert consensus^27^ on LAAO at 39.0%. At 30 days after the procedure, the rate of the composite end point of death, stroke, and systemic embolism was 0.52%, and the rate of any life-threatening or major bleeding was 1.23%. There was no significant association of the type of anesthesia (general vs local), modality of image guidance (TEE, ICE, or fluoroscopy), or isolated vs combination procedures with the procedural success, thrombotic events, or bleeding events at 30 days. Procedural success and rates of life-threatening or major bleeding at 30 days were significantly lower in centers in performing 40 or more procedures per year vs those performing fewer than 40 procedures per year.

A comparison of patient and procedural characteristics and clinical outcomes between the current and previous studies is summarized in Table 4. There was heterogeneity between studies; nevertheless, the RECORD population had numerically similar ischemic and bleeding risks. While the mean CHADS_2_ score in the RECORD study was lower than that in the EWOLUTION^12^ and Prospective Randomized Evaluation of the WATCHMAN LAA Closure Device In Patients With Atrial Fibrillation Versus Long Term Warfarin Therapy (PREVAIL) trials,^4^ it was higher than that reported in the PROTECT-AF trial.^2^ Similarly, the HAS-BLED score of the RECORD population was lower than that of the National Cardiovascular Data Registry (NCDR)^9^ but higher than the EWOLUTION,^12^ Continued Access to PROTECT-AF (CAP),^22^ and Continued Access to PREVAIL (CAP2) studies.^5^ Among the LAAO registries conducted in Europe or US, the mean age of patients enrolled ranged from 72 to 76 years; whereas in the RECORD study and other studies conducted in Asia, the mean age of patients was between 65 and 71 years (eTable 14 in the Supplement). The different stroke and bleeding risks and adherence to OAC among participants might have led to a region-specific preference for receiving an LAAO treatment. In previous studies, implantation success was achieved in 88.1% to 99.1% of procedures^2,4,5,8,9,10,11,12^; in the RECORD study, the device success rate was 99.5%, and the procedure success rate was 97.9%. Overall, these results indicate that in the RECORD population, LAAO using the WATCHMAN device had high implantation success rates (ie, device success) and low rates of periprocedural complications (ie, procedural success).

**Table 4.** Comparison With Previous Studies

Characteristic	No. (%)									
	Amplatzer		WATCHMAN							
	ACP (cardiac plug)^11^	Amulet^8^	PROTECT-AF^2^	PREVAIL^4^	CAP^5,22^	CAP2^5^	EWOLUTION^12^	Postapproval registry^10^	NCDR^9^	RECORD
**Study characteristics**										
LAAO procedures, No.	1047	1088	463	269	566	578	1025	3822	38 158	3096
Enrollment period, year	2008.12-2013.12	2015.06-2016.09	2005.02-2008.06	2010.11-2013.01	2008.08.07-2010.06.30	2012.09.25-2014.03.21	2013.10-2015.05	2015.03-2016.02	2016.01-2018.12	2019.04-2020.10
Participating centers, No. (country or region)	22 (Greece)	61 (US)	59 (US and Europe)	50 (US)	26 (US and Europe)	48 (US)	47 (US and Europe)	(US)	495 (US)	39 (China)
**Baseline patient characteristics**										
Race and ethnicity										
Asian	NA	NA	4 (0.9)	1 (0.4)	9 (1.6)	4 (0.7)	NA	NA	621 (1.6)	3096 (100.0)
Black	NA	NA	6 (1.3)	6 (2.2)	11 (1.9)	7 (1.2)	NA	NA	1768 (4.6)	0
White	NA	NA	425 (91.8)	253 (94.1)	520 (91.9)	545 (94.1)	NA	NA	35 345 (92.6)	0
Others^a^	NA	NA	28 (6.0)	9 (3.3)	26 (4.6)	23 (4.0)	NA	NA	424 (1.2)	0
Age, mean (SD), y	75 (8.0)	75 (9.0)	72 (8.8)	74 (7.4)	74 (8.3)	75 (8.0)	73 (9.0)	NA	76 (8.1)	69 (9.0)
Sex										
Women	399 (38.1)	381 (35.0)	137 (29.6)	87 (32.3)	195 (34.5)	229 (39.6)	411 (40.1)	NA	15 690 (41.1)	1314 (42.4)
Men	648 (61.9)	707 (65.0)	326 (70.4)	182 (67.7)	371 (65.5)	349 (60.4)	614 (59.9)	NA	22 468 (58.9)	1782 (57.6)
Diabetes	306 (29.0)	NA	113 (24.4)	91 (33.8)	141 (24.9)	194 (33.7)	304 (29.7)	NA	14 396 (37.7)	720 (23.3)
Previous Stroke										
Ischemic stroke or TIA	404 (38.6)	424 (39.0)	82 (17.7)	74 (27.5)	172 (30.4)	167 (29.0)	312 (30.5)	NA	15 988 (41.9)	1380 (44.7)
Hemorrhagic stroke	NA	NA	NA	NA	NA	NA	155 (15.1)	NA	NA	107 (3.5)
Hypertension	909 (86.8)	914 (84.0)	413 (89.2)	238 (88.5)	503 (89.0)	533 (92.5)	885 (86.4)	NA	35 148 (92.1)	2130 (69.8)
Coronary artery disease	367 (35.1)	NA	NA	NA	241 (42.6)	246 (42.6)	NA	NA	18 126 (47.5)	879 (28.5)
Vascular disease	NA	NA	NA	NA	255 (45.1)	265 (45.8)	429 (41.9)	NA	16 767 (43.9)	1681 (54.5)
Congestive heart failure	274 (26.2)	NA	124 (26.8)	63 (23.4)	108 (19.1)	156 (27.1)	350 (34.2)	NA	14 266 (37.4)	462 (15.0)
LVEF	NA	NA	57.3 (9.7)	55.4 (10.0)	56.5 (8.9)	56.3 (9.3)	NA	NA	NA	60.0 (8.3)
Classification of AF										
Paroxysmal	453 (43.3)	NA	200 (43.2)	131 (48.7)	242 (42.8)	309 (53.5)	NA	NA	19 800 (51.9)	1249 (40.3)
Persistent	43 (4.1)	NA	97 (21.0)	85 (31.6)	171 (30.2)	148 (25.6)	NA	NA	8056 (21.1)	1277 (41.2)
Long-standing persistent or permanent	594 (57.0)	NA	160 (34.6)	42 (15.6)	136 (24.0)	83 (14.4)	NA	NA	10 135 (26.6)	570 (18.4)
CHADS_2_ score	2.8 (1.3)	NA	2.2 (1.2)	2.6 (1.0)	2.5 (1.2)	2.7 (1.1)	2.8 (1.3)	NA	NA	2.3 (1.4)
CHA_2_DS_2_-VASc score	4.5 (1.6)	4.2 (1.6)	3.4 (1.5)	3.8 (1.2)	3.9 (1.5)	4.5 (1.3)	4.5 (1.6)	NA	4.6 (1.5)	4.0 (1.8)
HAS-BLED score	3.1 (1.2)	3.3 (1.1)	NA	NA	2.3 (1.1)	2.0 (0.9)	2.3 (1.2)	NA	3.0 (1.1)	2.4 (1.2)
**Events within 7 d after LAAO or during hospitalization**										
Devices used per procedure, mean, No.	NA	NA	1.6	1.5	1.4	NA	1.1	1.4	NA	1.0
Implantation success^b^	1019 (97.3)	1078 (99.1)	408 (90.9)	252 (95.1)	534 (94.4)	548 (94.8)	1004 (98.3)	3653 (95.6)	35 540 (98.3)	3082 (99.5)
Death	6 (0.6)	3 (0.3)	0	0	0	1 (0.2)	4 (0.4)	4 (0.1)	74 (0.2)	2 (0.1)
Stroke or TIA	13 (1.2)	0(0)	5 (1.1)	1 (0.4)	0	2 (0.4)	1 (0.1)	3 (0.1)	66 (0.2)	8 (0.3)
Pericardial tamponade	13 (1.2)	10 (0.9)	20 (4.3)	5 (1.9)	8 (1.4)	11 (1.9)	3 (0.3)	39 (1.0)	528 (1.4)	11 (0.3)
Treated with pericardiocentesis	NA	NA	13 (2.8)	4 (1.5)	7 (1.2)	NA	2 (0.2)	24 (0.6)	437 (1.2)	10 (0.4)
Treated surgically	NA	NA	7 (1.5)	1 (0.4)	1 (0.2)	NA	1 (0.1)	12 (0.3)	91 (0.3)	1 (<0.1)
Resulted in death	2 (0.2)	NA	0	0	0	0	0	3 (0.1)	0	0
Pericardial effusion, no intervention	NA	8 (0.7)	4 (0.9)	0	5 (0.9)	3 (0.5)	4 (0.4)	11 (0.3)	93 (0.3)	20 (0.6)
Device embolization	8 (0.8)	2 (0.2)	3 (0.6)	2 (0.7)	1 (0.2)	0	2 (0.2)	9 (0.2)	30 (0.1)	0
**Postoperative anticoagulation medication**										
Warfarin only	167 (16.0)	18 (1.7)	463 (100.0)	269 (100.0)	566 (100.0)	578 (100.0)	NR (16.0)	NA	NA	371 (12.0)
OAC only	14 (1.3)	31 (28.5)	0	0	0	0	NR (11.0)	NA	NA	2056 (66.7)
DAPT only	164 (15.7)	619 (56.9)	0	0	0	0	NR (60.0)	NA	NA	154 (5.0)
SAPT only	363 (34.7)	242 (22.2)	0	0	0	0	NR (7.0)	NA	NA	50 (1.6)
No treatment	87 (8.3)	23 (2.1)	NA	NA	NA	NA	NA	NA	NA	5 (0.1)
Others^c^	252 (24.1)	155 (14.2)	NA	NA	NA	NA	NR (6.0)	NA	NA	446 (14.4)

Abbreviations: CAP, Continued Access to PROTECT-AF; CAP2, Continued Access to PREVAIL; CHADS_2_, congestive heart failure, hypertension, age older than 75 years, diabetes, prior stroke or transient ischemic attack; CHA_2_DS_2_-VASc, congestive heart failure, hypertension, age 75 years or older, diabetes, stroke or transient ischemic attack, vascular disease, age 65 to 74 years, sex category; DAPT, dual antiplatelet therapy; HAS-BLED, hypertension, abnormal kidney or liver function, stroke, bleeding history or predisposition, labile international normalized ratio, elderly (ie, aged >65 years), and using drugs or alcohol concomitantly; LAAO, left atrial appendage occlusion; NA, not applicable; NCDR, National Cardiovascular Data Registry; NR, not reported; OAC, oral anticoagulant; PREVAIL, Prospective Randomized Evaluation of the WATCHMAN LAA Closure Device In Patients With Atrial Fibrillation Versus Long Term Warfarin Therapy; RECORD, Registry to Evaluate Chinese Real-World Clinical Outcomes in Patients With AF Using the WATCHMAN Left Atrial Appendage Closure Technology; SAPT, single antiplatelet therapy.

^a^ Other races and ethnicities included American Indian, Alaskan Native, Hawaiian, Hispanic, Latin American, and Pacific Islander.

^b^ Implantation success was defined as the delivery and release of the device into the LAA.

^c^ Other medications included acetylsalicylic acid with clopidogrel, warfarin, OAC, or low molecular–weight heparin; clopidogrel with warfarin or OAC; low molecular–weight heparin, triple therapy, and unknown.

The optimal technique for implanting an LAAO device remains unclear, and consequently, techniques vary among centers and surgeons. The standard method of intraprocedural imaging during LAAO is TEE; however, ICE or fluoroscopy are 2 suitable alternatives that also facilitate the use of local rather than general anesthesia.^1^ Our results support previous nonrandomized series and meta-analyses reporting that ICE-guided LAAO was feasible and as effective as TEE^28,29^; however, the sample size of ICE-guided LAAO was small in our analysis. The European Heart Rhythm Association and European Association of Percutaneous Cardiovascular Interventions expert consensus statement on catheter-based LAAO^1^ suggested that procedural imaging using only fluoroscopy should be reserved for exceptional circumstances and performed only by experts. Moreover, the recently presented Bern registry^30^ suggested that the use of TEE or ICE to guide LAAO was associated with significantly lower risks of procedural complications compared with fluoroscopy alone. In the RECORD study, the rate of intraprocedural imaging using only fluoroscopy increased when centers were performing more cases annually. After adjusting for confounding factors, the rates of procedural success and 30-day ischemic or bleeding end points did not differ significantly among procedures using TEE, ICE, or fluoroscopy alone. Our results showed that procedural imaging using only fluoroscopy, which was primarily conducted in experienced high-volume centers, was safe at 30 days; however, with current available evidence, we do not support the routine use of fluoroscopy guidance alone during LAAO, and its efficacy still needs to be carefully evaluated in further studies before definitive recommendations are possible.

Combining an LAAO implant with a catheter ablation for AF has been shown to be feasible and safe in small-scale registries^31,32^ and a meta-analysis that included 1154 patients from 18 observational studies.^33^ On the other hand, a study by Turagam et al^34^ suggested that electrical isolation after LAAO could be difficult and, when attempted, could result in an increased risk of short-term peridevice leak and recurrence of atrial tachyarrhythmias or AF. Unfortunately, specific information regarding combined AF ablation is not available from prior pivotal randomized clinical trials (an exclusion criterion in both the PROTECT AF^2^ and PREVAIL^4^ trials) or large-scale observational studies.^5,8,9,10,11,12,22^ In the RECORD study, we found that 41.9% of LAAO procedures were combined with an AF ablation, with this high percentage likely to be because 80.5% of surgeons were electrophysiologists. Reassuringly, we found that these combined procedures were not associated with increased short-term adverse event rates; their long-term outcomes, such as the recurrence rates of atrial tachyarrhythmias or AF, will be assessed in a longer follow-up of the RECORD study.

There was no specific recommendation for the antithrombotic medications after LAAO in the ACC guidelines for AF.^14^ Whereas the ESC guidelines for AF^13^ recommended using aspirin plus clopidogrel, and the CSC expert consensus^27^ suggests monotherapy with OAC or NOAC in patients with high bleeding risk (HAS-BLED ≥3) during the first month after LAAO. For patients with a low bleeding risk (HAS-BLED <3), both the ESC and CSC recommend aspirin plus OAC or NOAC. Notably, these recommendations only have limited supportive evidence, since they have not been formally tested in randomized clinical trials.^13,27,35^ In the RECORD study, two-thirds of discharged patients were using NOAC monotherapy, such that adherence rates to post-LAAO medication recommendations from the ESC and the CSC were low overall. In an upcoming report 1-year outcomes of the RECORD study, we will explore the association of regimen of immediate NOAC monotherapy after LAAO with clinical outcomes.

Operators who perform LAAO procedures come from a variety of backgrounds, including interventional cardiology (adult or pediatric), electrophysiology, and cardiac surgery. Data showing the number of procedures required for a surgeon to become fully competent to perform the procedure, and the number required to maintain competence are limited.^36^ Although it has been suggested that LAAO with WATCHMAN is safe among inexperienced surgeons,^10^ a single-center all-comer registry investigating the learning curve with the device concluded that the threshold for competency in terms of procedure time, fluoroscopy time, and contrast volume was 30 patients.^37^ The consensus statement from the Society for Cardiovascular Angiography and Interventions, ACC, and Heart Rhythm Society^36^ recommended that institutions perform more than 50 structural or left-sided catheter ablations per year, with at least 25 involving a transseptal puncture through an intact septum in the year leading to program initiation and per year thereafter. In a study using the Nationwide Readmission Database from the US, Vuddandi et al^38^ found that when compared across strata based on hospital LAAO procedural volume(low: <20 procedures per year; intermediate: 21-50 procedures per year; and high: >50 procedures per year), there was no difference in inpatient complications. In this study, we established a threshold of 40 procedures per year for defining an experienced center, as once this level of skill and competence is achieved and maintained, it is difficult to improve on the rate of procedural success.

### Limitations

This study has some limitations. Imbalances exist among the subgroups assessed, such as procedural configurations. Although statistical adjustments were made to try to estimate the true differences between groups, the inability to eliminate the impact of unmeasurable confounders produces bias that cannot be adjusted for. Given the observational nature of the study, all reported results should be strictly considered as exploratory and hypothesis-generating only. While the RECORD registry only included 39 Chinese centers, the Japanese and Korean population and other East Asian ethnic groups were not represented in this study; therefore, any extrapolation of these results to the overall East Asian population should be done with caution. Although the clinical adverse events were adjudicated by clinical event committee, the status of LAA sealing immediately after procedures was not centrally adjudicated. The site-reported LAA sealing evaluated immediately after procedures might be biased toward a higher complete sealing rate. The site-reported LAA sealing status will be compared with computed tomography angiography or TEE follow-up in a future report of the RECORD study.

## Conclusions

This cohort study of the RECORD study conducted among patients undergoing LAAO in China found that the WATCHMAN percutaneous LAAO device had a high rate of procedural success and low rates of periprocedural complications and short-term ischemic and bleeding events. There was a significant relationship between annual case volume and outcomes among centers.

## Notes

Supplement. **eFigure 1.** Study Flowchart **eFigure 2.** CHA_2_DS_2_-VASc and HAS-BLED Score and the Proportions of Individual Components of the Scores **eFigure 3.** Proportion of Anesthesia and Intraprocedural Imaging Guidance by Center Volume **eFigure 4.** Device, Technical, and Procedural Success According to the Munich Consensus **eFigure 5.** Procedure Success Rates According to the Volume of Centers **eFigure 6.** Procedure Success According to the Expertise of Operators **eTable 1.** Post-LAAO Medication **eTable 2.** Distribution of Post-LAAO Novel Oral Anticoagulation Medication Doses **eTable 3.** 2019 Chinese Society of Cardiology (CSC) Expert Consensus Statement on Left Atrial Appendage Closure in the Prevention of Stroke in Patients With Atrial Fibrillation **eTable 4.** Clinical Events According to the ESC AF Guideline and CSC Expert Consensus on LAAO **eTable 5.** Medication According to the CSC Expert Consensus on LAAO **eTable 6.** Baseline Characteristics According to the Volume of Centers **eTable 7.** Procedural Configurations According to the Volume of Centers **eTable 8.** Subgroups Analyses by the Composite End Point of Death, Stroke, and Systemic Embolism **eTable 9.** Subgroups Analyses by the Composite End Point of Any Life-threatening or Major Bleeding Events **eTable 10.** Subgroups Analyses by the Composite End Point of Death, Stroke, Systemic Embolism, and Any Life-threatening or Major Bleeding Events **eTable 11.** Associations of Procedural Configurations With Outcomes **eTable 12.** Baseline Characteristics Patients Who Underwent of Noncombined vs Combined Radiofrequency Ablation and Cryoablation **eTable 13.** Clinical Events of Patients Who Underwent of Noncombined vs Combined Radiofrequency Ablation and Cryoablation at 30 Days After the Procedure **eTable 14.** LAAO Registries Conducted in Asia **eTable 15.** Clinical Events at 45 Days After LAOO
