# Supplementary material for: Periprocedural Outcomes Associated With Use of a Left Atrial Appendage Occlusion Device in China
Source: JAMA Netw Open. 2022 May 31;5(5):e2214594. doi: 10.1001/jamanetworkopen.2022.14594 (PMC9157261; doi:10.1001/jamanetworkopen.2022.14594)
Supplement: Supplement. — eFigure 1. Study Flowchart eFigure 2. CHA2DS2-VASc and HAS-BLED Score and the Proportions of Individual Components of the Scores eFigure 3. Proportion of Anesthesia and Intraprocedural Imaging Guidance by Center Volume eFigure 4. Device, Technical, and Procedural Success According to the Munich Consensus eFigure 5. Procedure Success Rates According to the Volume of Centers eFigure 6. Procedure Success According to the Expertise of Operators eTable 1. Post-LAAO Medication eTable 2. Distribution of Post-LAAO Novel Oral Anticoagulation Medication Doses eTable 3. 2019 Chinese Society of Cardiology (CSC) Expert Consensus Statement on Left Atrial Appendage Closure in the Prevention of Stroke in Patients With Atrial Fibrillation eTable 4. Clinical Events According to the ESC AF Guideline and CSC Expert Consensus on LAAO eTable 5. Medication According to the CSC Expert Consensus on LAAO eTable 6. Baseline Characteristics According to the Volume of Centers eTable 7. Procedural Configurations According to the Volume of Centers eTable 8. Subgroups Analyses by the Composite End Point of Death, Stroke, and Systemic Embolism eTable 9. Subgroups Analyses by the Composite End Point of Any Life-threatening or Major Bleeding Events eTable 10. Subgroups Analyses by the Composite End Point of Death, Stroke, Systemic Embolism, and Any Life-threatening or Major Bleeding Events eTable 11. Associations of Procedural Configurations With Outcomes eTable 12. Baseline Characteristics Patients Who Underwent of Noncombined vs Combined Radiofrequency Ablation and Cryoablation eTable 13. Clinical Events of Patients Who Underwent of Noncombined vs Combined Radiofrequency Ablation and Cryoablation at 30 Days After the Procedure eTable 14. LAAO Registries Conducted in Asia eTable 15. Clinical Events at 45 Days After LAOO [file jamanetwopen-e2214594-s001.pdf]

Supplemental Online Content

Su F, Gao C, Liu J, et al. Periprocedural outcomes associated with use of a left atrial appendage occlusion device in China. *JAMA Netw Open*. 2022;5(5):e2214594. doi:10.1001/jamanetworkopen.2022.14594

eFigure 1. Study Flowchart
eFigure 2. CHA_2DS_2-VASc and HAS-BLED Score and the Proportions of Individual Components of the Scores
eFigure 3. Proportion of Anesthesia and Intraprocedural Imaging Guidance by Center Volume
eFigure 4. Device, Technical, and Procedural Success According to the Munich Consensus
eFigure 5. Procedure Success Rates According to the Volume of Centers
eFigure 6. Procedure Success According to the Expertise of Operators
eTable 1. Post-LAAO Medication
eTable 2. Distribution of Post-LAAO Novel Oral Anticoagulation Medication Doses
eTable 3. 2019 Chinese Society of Cardiology (CSC) Expert Consensus Statement on Left Atrial Appendage Closure in the Prevention of Stroke in Patients With Atrial Fibrillation
eTable 4. Clinical Events According to the ESC AF Guideline and CSC Expert Consensus on LAAO
eTable 5. Medication According to the CSC Expert Consensus on LAAO
eTable 6. Baseline Characteristics According to the Volume of Centers
eTable 7. Procedural Configurations According to the Volume of Centers
eTable 8. Subgroups Analyses by the Composite End Point of Death, Stroke, and Systemic Embolism
eTable 9. Subgroups Analyses by the Composite End Point of Any Life-threatening or Major Bleeding Events
eTable 10. Subgroups Analyses by the Composite End Point of Death, Stroke, Systemic Embolism, and Any Life-threatening or Major Bleeding Events
eTable 11. Associations of Procedural Configurations With Outcomes
eTable 12. Baseline Characteristics Patients Who Underwent of Noncombined vs Combined Radiofrequency Ablation and Cryoablation
eTable 13. Clinical Events of Patients Who Underwent of Noncombined vs Combined Radiofrequency Ablation and Cryoablation at 30 Days After the Procedure
eTable 14. LAAO Registries Conducted in Asia
eTable 15. Clinical Events at 45 Days After LAAO

This supplemental material has been provided by the authors to give readers additional information about their work.

© 2022 Su F et al. *JAMA Network Open.*

eFigure 1. Study Flowchart

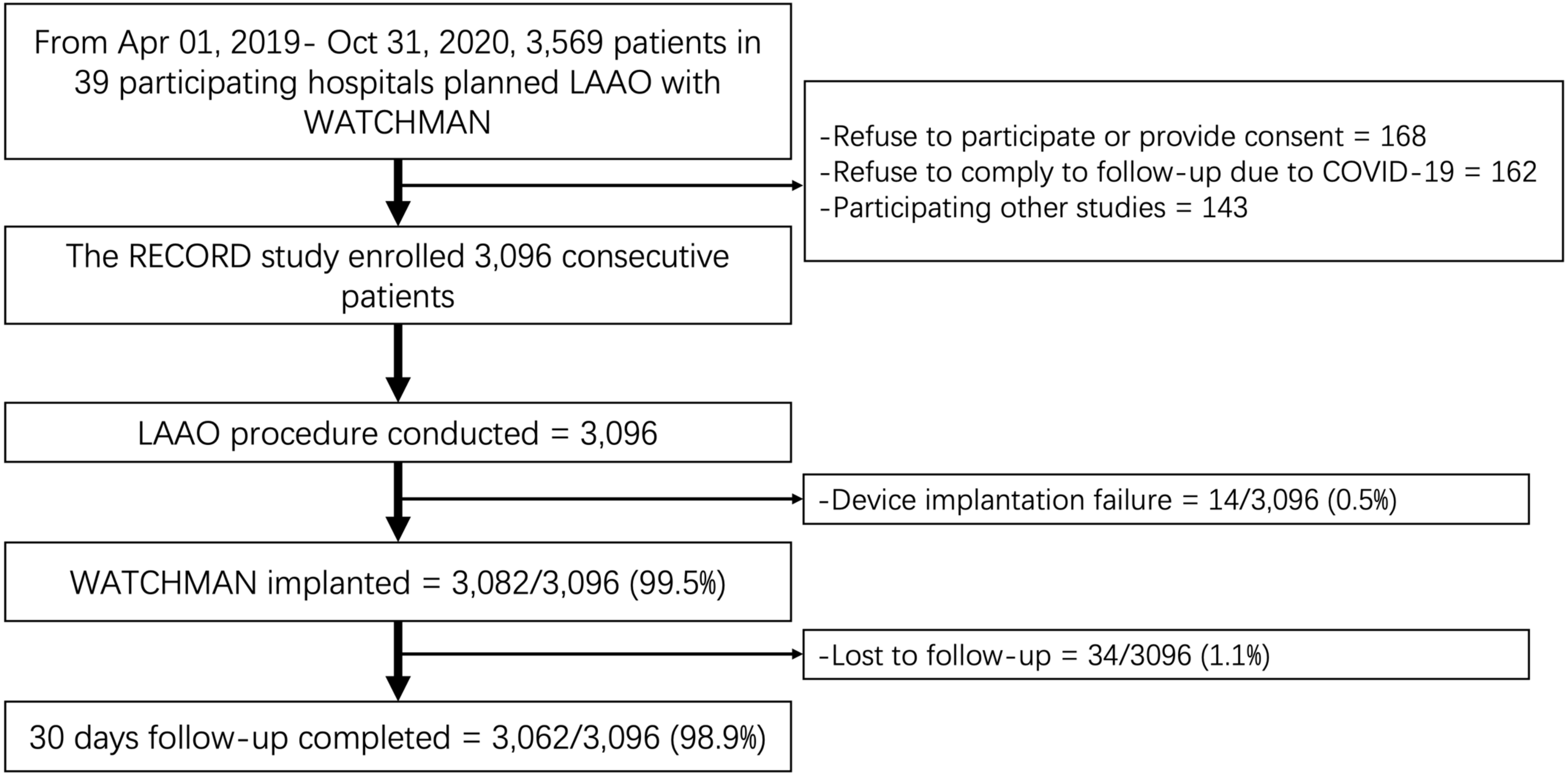

© 2022 Su F et al. *JAMA Network Open.*

eFigure 2. CHA_2DS_2-VASc and HAS-BLED Score and the Proportions of Individual Components of the Scores

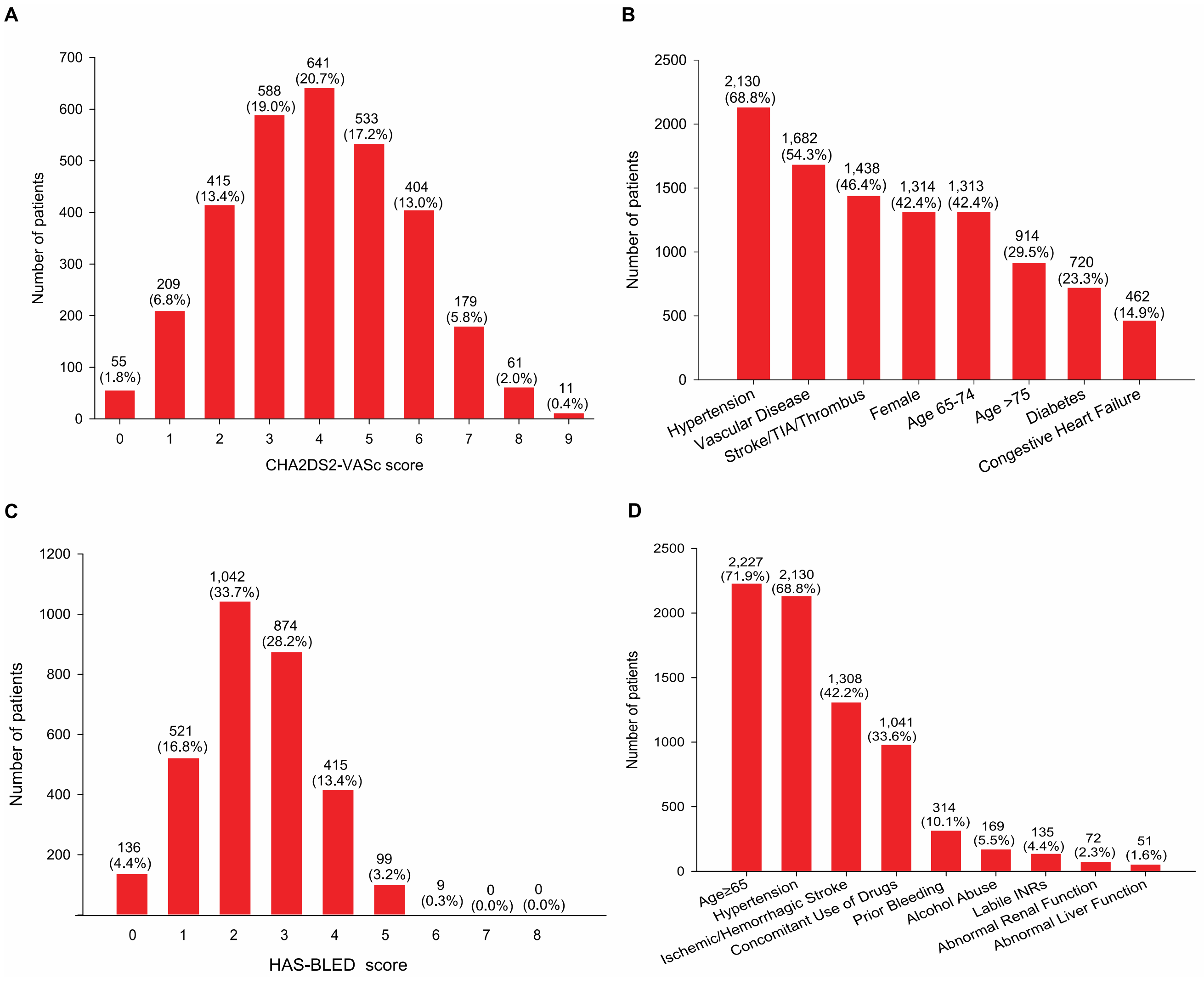

© 2022 Su F et al. *JAMA Network Open.*

eFigure 3. Proportion of Anesthesia and Intraprocedural Imaging Guidance by Center Volume

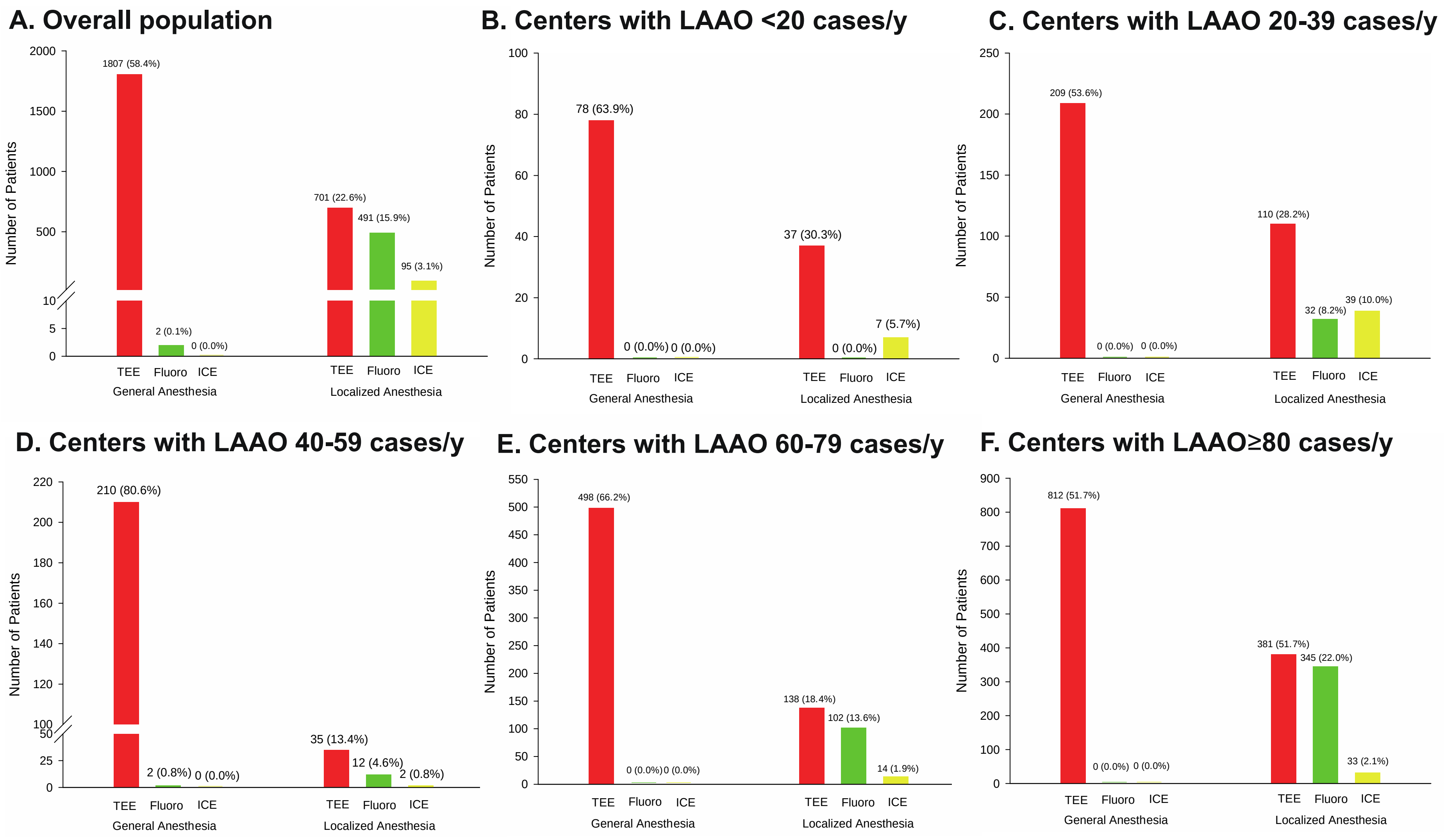

© 2022 Su F et al. *JAMA Network Open.*

eFigure 4. Device, Technical, and Procedural Success According to the Munich Consensus

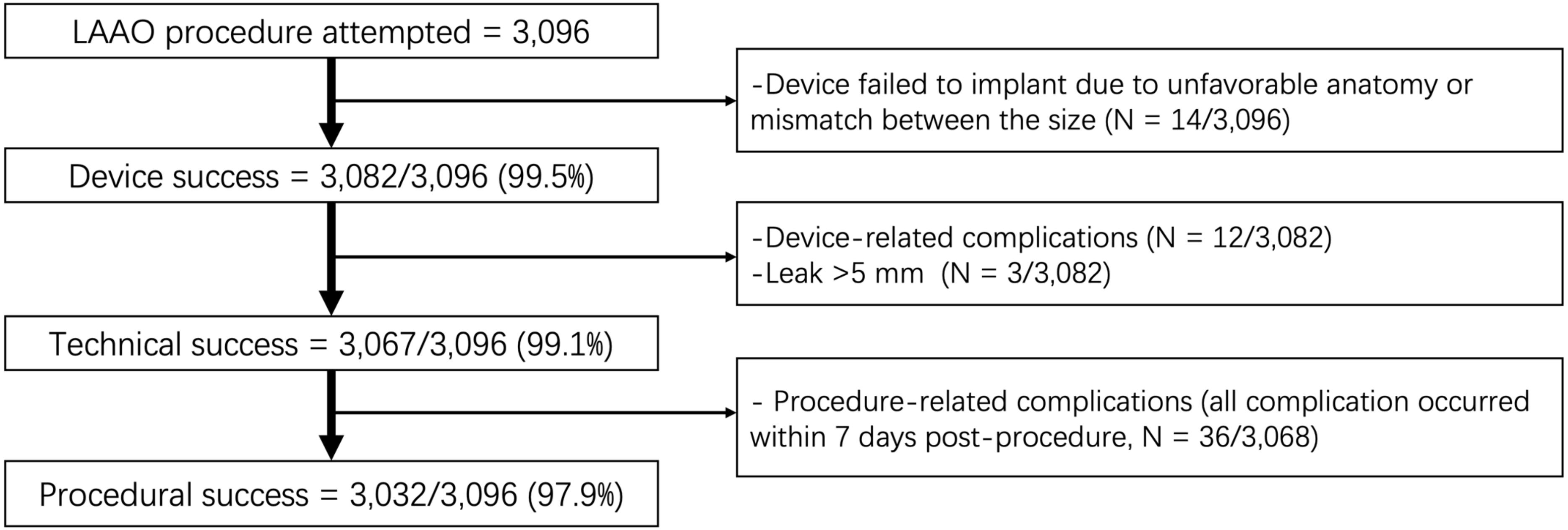

© 2022 Su F et al. *JAMA Network Open.*

eFigure 5. Procedure Success Rates According to the Volume of Centers

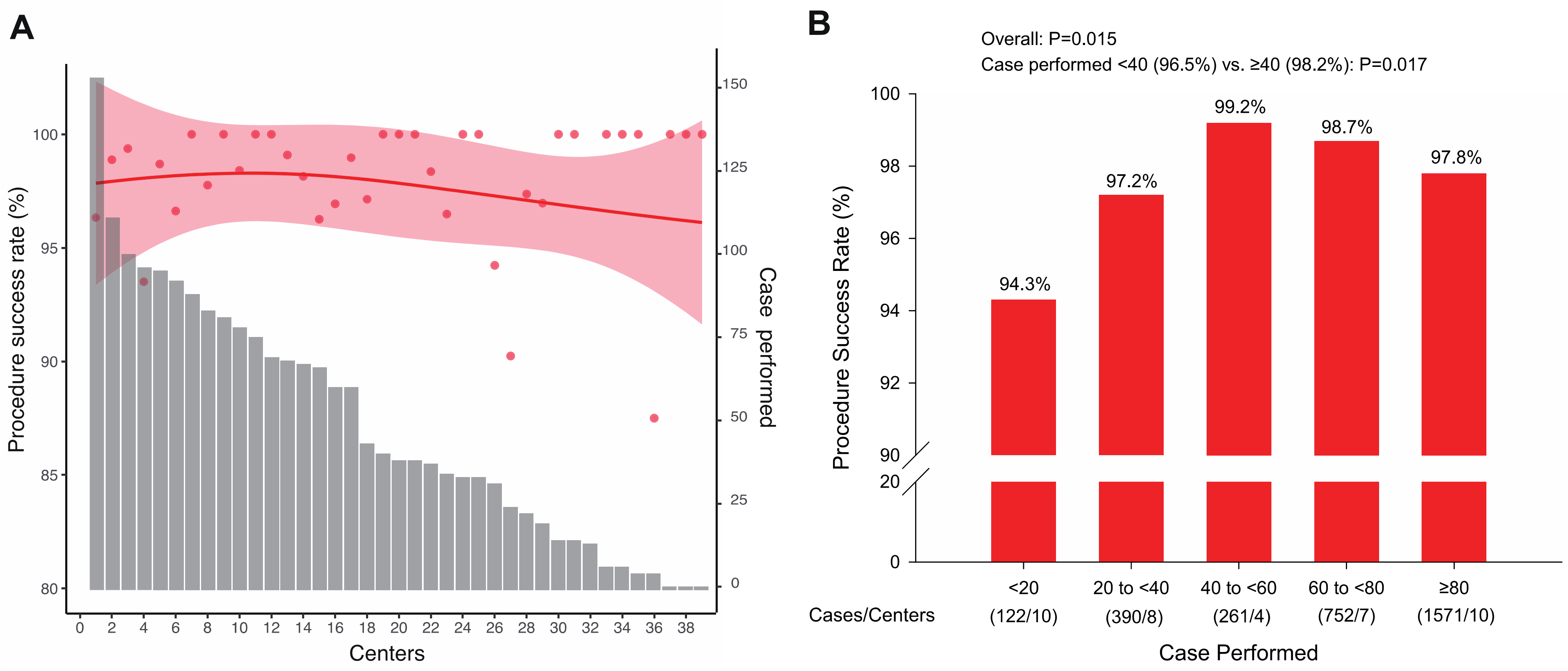

© 2022 Su F et al. *JAMA Network Open.*

eFigure 6. Procedure Success According to the Expertise of Operators

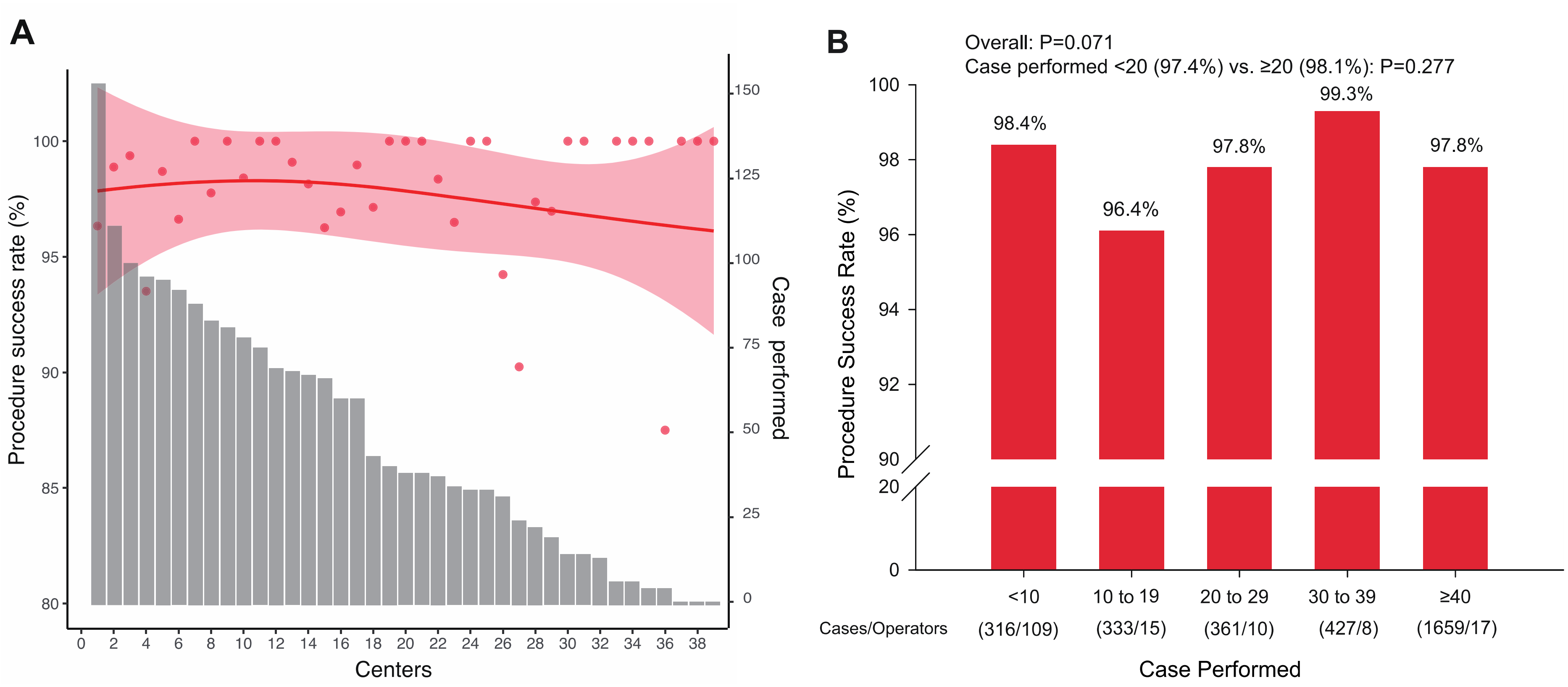

* The procedure success rates of operators who conducted less than 5 cases annually were combined

© 2022 Su F et al. *JAMA Network Open.*

eTable 1. Post-LAAO Medication

Overall	Warfarin plus Aspirin/P2Y12 inhibitor	NOAC plus Aspirin/P2Y12 inhibitor	Warfarin monotherapy	NOAC monotherapy	DAPT	SAPT	Others
	N=35 (1.1%)	N=390 (12.7%)	N=371 (12.0%)	N=2,056 (66.7%)	N=154 (5.0%)	N=50 (1.6%)	N=26 (0.8%)
Death, stroke, embolism	0 (0.0)	2 (0.5)	1 (0.3)	7 (0.3)	1 (0.6)	3 (6.0)	2 (7.7)
Any life-threatening or major bleeding	1 (2.9)	3 (0.8)	2(0.5)	22 (1.1)	3 (1.9)	3 (6.0)	3 (11.5)
Death, stroke, embolism and any life-threatening or major bleeding	1 (2.9)	5 (1.3)	3 (0.8)	27 (1.3)	4 (2.6)	4 (8.0)	5 (19.2)
HAS-BLED≥3	Warfarin plus Aspirin/P2Y12 inhibitor	NOAC plus Aspirin/P2Y12 inhibitor	Warfarin monotherapy	NOAC monotherapy	DAPT	SAPT	Others
	N=16 (1.1%)	N=215 (15.5%)	N=191 (13.7%)	N=838 (60.2%)	N=94 (6.7%)	N=24 (1.7%)	N=13 (0.9%)
Death, stroke, embolism	0 (0.0)	0 (0.0)	1 (0.5)	5 (0.6)	1 (1.1)	3 (12.5)	2 (15.4)
Any life-threatening or major bleeding	1 (6.3)	3 (1.4)	1 (0.5)	11 (1.3)	0 (0.0)	3 (12.5)	1 (7.7)
Death, stroke, embolism and any life-threatening or major bleeding	1 (6.3)	3 (1.4)	2 (1.0)	15 (1.8)	1 (1.1)	4 (16.7)	3 (23.1)
HAS-BLED<3	Warfarin plus Aspirin/P2Y12 inhibitor	NOAC plus Aspirin/P2Y12 inhibitor	Warfarin monotherapy	NOAC monotherapy	DAPT	SAPT	Others
	N=19 (1.1%)	N=175 (10.3%)	N=180 (10.6%)	N=1218 (72.0%)	N=60 (3.6%)	N=26 (1.5%)	N=13 (0.8%)
Death, stroke, embolism	0 (0.0)	2 (1.1)	0 (0.0)	2 (0.2)	0 (0.0)	0 (0.0)	0 (0.0)
Any life-threatening or major bleeding	0 (0.0)	0 (0.0)	1 (0.6)	11 (0.9)	3 (5.0)	0 (0.0)	2 (15.4)

© 2022 Su F et al. *JAMA Network Open.*

Death, stroke, embolism and any life-threatening or major bleeding	0 (0.0)	2 (1.1)	1 (0.6)	12 (1.0)	3 (5.0)	0 (0.0)	2 (15.4)

© 2022 Su F et al. *JAMA Network Open.*

eTable 2. Distribution of Post-LAAO Novel Oral Anticoagulation Medication Doses

Rivaroxaban	2.5mg	5mg	7.5mg	10mg	15mg	20mg	25mg	Total
Rivaroxaban	2 (0.1)	20 (1.1)	2 (0.1)	483 (27.2)	693 (39.0)	255 (14.4)	0 (0.0)	1,455 (81.9)
Rivaroxaban + Aspirin	0 (0.0)	0 (0.0)	0 (0.0)	31 (1.7)	29 (1.6)	62 (3.5)	0 (0.0)	122 (10.2)
Rivaroxaban + Clopidogrel	1 (0.1)	1 (0.1)	0 (0.0)	67 (3.8)	98 (5.5)	14 (0.8)	1 (0.1)	182 (10.2)
Rivaroxaban + Ticagrelor	0 (0.0)	1 (0.1)	0 (0.0)	1 (0.1)	2 (0.1)	0 (0.0)	0 (0.0)	4 (0.2)
Rivaroxaban + DAPT	5 (0.3)	0 (0.0)	0 (0.0)	4 (0.2)	5 (0.3)	0 (0.0)	0 (0.0)	14 (0.8)
Total	8 (0.5)	22 (1.2)	2 (0.1)	586 (33.0)	827 (46.5)	331 (18.6)	1 (0.1)	1,777(100.0)

© 2022 Su F et al. *JAMA Network Open.*

eTable 2. Continued

Dabigatran	110mg	150mg	Total
Dabigatran	516 (74.4)	94 (13.5)	610 (87.9)
Dabigatran + Aspirin	30 (4.3)	1 (0.1)	31 (4.5)
Dabigatran + Clopidogrel	48 (6.9)	3 (0.4)	51 (7.3)
Dabigatran + Aspirin + Clopidogrel	2 (0.3)	0 (0.0)	2 (0.3)
Total	596 (85.9)	98 (14.1)	694 (100.0)

© 2022 Su F et al. *JAMA Network Open.*

eTable 3. 2019 Chinese Society of Cardiology (CSC) Expert Consensus Statement on Left Atrial Appendage Closure in the Prevention of Stroke in Patients With Atrial Fibrillation

Post-LAAO within 3 months	Recommendation	Level
GFR≥30 ml/min & HAS-BLED<3	NOAC + Aspirin/Clopidogrel<br>or<br>Warfarin (INR 2 -3) + Aspirin/Clopidogrel	I
GFR≥30 ml/min & HAS-BLED≥3	NOAC monotherapy<br>or<br>Warfarin monotherapy (INR 2 -3)	I
GFR<30 ml/min & HAS-BLED<3	Warfarin (INR 2 -3) + Aspirin	I
GFR<30 ml/min & HAS-BLED≥3	Aspirin + Clopidogrel<br>or<br>Warfarin monotherapy (INR 2 -3)	I
Aspirin or Clopidogrel monotherapy		III
Discontinue antiplatelet or anticoagulation		III

© 2022 Su F et al. *JAMA Network Open.*

eTable 4. Clinical Events According to the ESC AF Guideline and CSC Expert Consensus on LAAO

**ESC AF guideline (2020)**						
**HAS-BLED≥3**	Compliant	Non-compliant	Univariate		Multivariate	
	94/1,391	1,297/1,391	OR (95%CI)	P	OR (95%CI)	P
Death, stroke, systemic embolism	1 (1.06)	11 (0.85)	0.80 (0.10-6.23)	0.827	0.90 (0.11-7.38)	0.923
Any life-threatening or major bleeding	0 (0.00)	20 (1.54)	-	-	-	-
Death, stroke, embolism and any life-threatening or major bleeding	1 (1.06)	28 (2.16)	2.05 (0.28-15.25)	0.480	2.37 (0.31-17.99)	0.403
**HAS-BLED<3**	Compliant	Non-compliant	Univariate		Multivariate	
	98/1,691	1,593/1,691	OR (95%CI)	P	OR (95%CI)	P
Death, stroke, systemic embolism	0 (0.00)	4 (0.25)	-	-	-	-
Any life-threatening or major bleeding	0 (0.00)	17 (1.07)	-	-	-	-
Death, stroke, embolism and any life-threatening or major bleeding	0 (0.00)	20 (1.26)	-	-	-	-
**Chinese expert consensus (2019)**						
**HAS-BLED≥3**	Compliant	Non-compliant	Univariate		Multivariate	
	1,008/1,391	383/1,391	OR (95%CI)	P	OR (95%CI)	P
Death, stroke, systemic embolism	6 (0.60)	6 (1.57)	2.66 (0.85-8.29)	0.092	2.88 (0.90-9.29)	0.076
Any life-threatening or major bleeding	12 (1.19)	8 (2.09)	1.77 (0.72-4.37)	0.215	2.11 (0.83-5.32)	0.116
Death, stroke, embolism and any life-threatening or major bleeding	17 (1.69)	12 (3.13)	1.89 (0.89-3.99)	0.097	2.21 (1.02-4.77)	0.045
**HAS-BLED<3**	Compliant	Non-compliant	Univariate		Multivariate	
	194/1,691	1,497/1,691	OR (95%CI)	P	OR (95%CI)	P
Death, stroke, systemic embolism	2 (1.03)	2 (0.13)	-	-	-	-
Any life-threatening or major bleeding	0 (0.00)	17 (1.14)	-	-	-	-
Death, stroke, embolism and any life-threatening or major bleeding	2 (1.03)	18 (1.20)	1.17 (0.27-5.07)	0.836	1.14 (0.25-5.13)	0.864

Adjusted to type of anesthesia, type of guidance, combined procedure, the volume of centers, previous stroke, and bleeding history

© 2022 Su F et al. *JAMA Network Open.*

eTable 5. Medication According to the CSC Expert Consensus on LAAO

CSC expert consensus (2019)	Total	Warfarin plus Aspirin/ P2Y12 inhibitor	NOAC plus Aspirin/ P2Y12 inhibitor	Warfarin monotherapy	NOAC monotherapy	DAPT	SAPT	Others
HAS-BLED≥3 Compliance	N=1008	N=0	N=0	N=191	N=810	N=7	N=0	N=0
Death, stroke, embolism	6 (0.60)	-	-	1 (0.5)	5 (0.6)	0 (0.0)	-	-
Any life-threatening or major bleeding	12 (1.19)	-	-	1 (0.5)	11 (1.4)	0 (0.0)	-	-
Death, stroke, embolism and any life-threatening or major bleeding	17 (1.69)	-	-	2 (1.0)	15 (1.9)	0 (0.0)	-	-
HAS-BLED≥3 Non-compliance	N=383	N=16	N=215	N=0	N=28	N=87	N=24	N=13
Death, stroke, embolism	6 (1.57)	0 (0.0)	0 (0.0)	-	0 (0.0)	1 (1.1)	3 (12.5)	2 (15.4)
Any life-threatening or major bleeding	8 (2.09)	1 (6.3)	3 (1.4)	-	0 (0.0)	0 (0.0)	3 (12.5)	1 (7.7)
Death, stroke, embolism and any life-threatening or major bleeding	12 (3.13)	1 (6.3)	3 (1.4)	-	0 (0.0)	1 (1.1)	4 (16.7)	3 (23.1)
HAS-BLED<3 Compliance	N=194	N=19	N=175	N=0	N=0	N=0	N=0	N=0
Death, stroke, embolism	2 (1.03)	0 (0.0)	2 (1.1)	-	-	-	-	-
Any life-threatening or major bleeding	0 (0.00)	0 (0.0)	0 (0.0)	-	-	-	-	-
Death, stroke, embolism and any life-threatening or major bleeding	2 (1.03)	0 (0.0)	2 (1.1)	-	-	-	-	-
HAS-BLED<3 Non-compliance	N=1497	N=0	N=0	N=180	N=1218	N=60	N=26	N=13
Death, stroke, embolism	2 (0.13)	-	-	0 (0.0)	2 (0.2)	0 (0.0)	0 (0.0	0 (0.0)

© 2022 Su F et al. *JAMA Network Open.*

Any life-threatening or major bleeding	17 (1.14)	-	-	1 (0.6)	11 (0.9)	3 (5.0)	0 (0.0)	2 (15.4)
Death, stroke, embolism and any life-threatening or major bleeding	18 (1.20)	-	-	1 (0.1)	12 (1.0)	3 (5.0)	0 (0.0)	2 (15.4)

© 2022 Su F et al. *JAMA Network Open.*

eTable 6. Baseline Characteristics According to the Volume of Centers

Characteristics/Volume of the centers	Centers with LAAO <20 cases/y (N=122)	Centers with LAAO 20-39 cases/y (N=390)	Centers with LAAO 40-59 cases/y (N=261)	Centers with LAAO 60-79 cases/y (N=752)	Centers with LAAO ≥80 cases/y (N=1,571)
**Age, yrs**	68.9±9.5	68.0±9.0	68.2±9.3	66.8±9.3	70.7±9.2
65-74 years	56 (45.0)	177 (45.4)	105 (40.2)	342 (45.5)	633 (40.3)
≥75y	37 (30.3)	92 (23.6)	67 (25.7)	154 (20.5)	564 (35.9)
**Male**	71 (58.2)	233 (59.7)	157 (60.2)	419 (55.7)	902 (57.4)
**Body mass index (BMI)**	25.1±3.6	24.9±3.1	24.6±3.1	25.1±3.5	24.7±2.5
**Heart rate, per/min**	83.5±23.8	81.8±20.5	79.4±21.8	84.4±21.8	81.1±19.9
**Diabetes**	31 (25.4)	89 (22.8)	67 (25.7)	148 (19.7)	385 (24.5)
**Cerebrovascular disease**	65 (53.3)	199 (51.0)	164 (62.8)	354 (47.1)	636 (40.5)
TIA	4 (5.8)	7 (3.5)	10 (5.9)	21 (5.9)	115 (17.8)
Ischemic stroke	60 (49.2)	186 (47.7)	153 (58.6)	327 (43.5)	544 (34.6)
Haemorrhagic stroke	9 (7.4)	19 (4.9)	9 (3.4)	25 (3.3)	45 (2.9)
**Hypertension**	84 (68.9)	243 (62.3)	204 (78.2)	504 (67.0)	1,095 (69.7)
**Coronary artery disease**	36 (29.5)	108 (27.7)	58 (22.2)	208 (27.7)	469 (29.9)
Previous PCI	12 (9.8)	34 (8.7)	29 (11.1)	46 (6.1)	206 (13.1)
Previous CABG	0 (0.0)	6 (1.5)	1 (0.4)	8 (1.1)	31 (2.0)
**Vascular disease**	66 (54.1)	230 (59.0)	138 (52.9)	381 (50.7)	867 (55.2)
**Current Smoker**	26 (21.3)	52 (13.3)	27 (10.3)	77 (10.2)	152 (9.7)
**Alcohol abuse**	13 (10.7)	12 (3.1)	12 (4.6)	54 (7.2)	78 (5.0.0)
**Chronic heart failure**	24 (19.7)	49 (12.6)	13 (5.0)	113 (15.0)	236 (16.7)
**LVEF**	59.8±7.5	59.6±8.8	59.2±7.8	59.8±8.6	60.4±8.2
**Abnormal thyroidal function**	5 (4.1)	25 (6.4)	7 (2.7)	38 (5.1)	58 (3.7)
**Abnormal renal function**	4 (3.3)	11 (2.8)	4 (1.5)	17 (2.3)	36 (2.3)
**Abnormal liver function**	3 (2.5)	11 (2.8)	5 (1.9)	6 (0.8)	26 (1.7)
**Bleeding history or predisposition**	24 (19.7)	43 (11.0)	18 (6.9)	79 (10.5)	150 (9.6)

© 2022 Su F et al. *JAMA Network Open.*

Previous gastrointestinal bleeding	5 (4.1)	8 (2.1)	1 (0.4)	16 (2.1)	43 (2.7)
**Concomitant use of drugs**					
**Classification of AF**	38 (31.2)	138 (35.4)	49 (18.8)	247 (32.9)	777 (49.5)
Paroxysmal	71 (58.2)	174 (44.6)	134 (51.3)	307 (40.9)	491 (37.6)
Persistent	13 (10.7)	78 (20.0)	78 (29.9)	198 (26.3)	203 (12.9)
Long-standing persistent (>1y)/Permanent	2.6±1.3	2.2±1.3	2.6±1.2	2.2±1.4	2.3±1.4
**$CHADS_2$ score**	4.3±1.6	3.9±1.7	4.2±1.6	3.8±1.8	4.0±1.9
**CHA_2DS_2-VASc score**	2.8±1.2	2.3±1.1	2.6±1.1	2.3±1.2	2.4±1.2
**HAS-BLED score**	6.9±2.5	6.4 ±2.9	7.1±2.7	6.0±3.1	6.1±2.9
ATRIA score	35 (28.7)	110 (28.2)	96 (36.8)	220 (29.3)	580 (36.9)

© 2022 Su F et al. *JAMA Network Open.*

eTable 7. Procedural Configurations According to the Volume of Centers

Characteristics/Volume of the centers	Centers with LAAO <20 cases/y	Centers with LAAO 20-39 cases/y	Centers with LAAO 40-59 cases/y	Centers with LAAO 60-79 cases/y	Centers with LAAO ≥80 cases/y
**Recaptured (≥2 times) before release**	10/122 (8.2)	13/390 (3.3)	3/261 (1.2)	79/752 (10.5)	153/1,571 (9.7)
**Device used**	130/122	395/390	261/261	789/752	1,630/1,571
**Device size**					
21 mm	6/120 (5.0)	30/ 389 (7.7)	14/260 (5.4)	48/747 (6.4)	90/1,566 (5.8)
24 mm	29/120 (24.2)	71/389 (18.3)	54/260 (20.8)	170/747 (22.8)	310/1,566 (19.8)
27 mm	43/120 (35.8)	109/389 (28.0)	71/260 (27.3)	238/747 (31.9)	457/1,566 (29.2)
30 mm	24/120 (20.0)	97/389 (24.9)	60/260 (23.1)	163/747 (21.8)	370/1,566 (23.6)
33 mm	19/120 (15.8)	82/389 (21.1)	61/260 (23.5)	132/747 (17.7)	339/1,566 (21.7)
**Residual peri-device leakage**					
Complete sealing	116/120 (96.7)	349/389 (89.7)	234/260 (90.0)	658/747 (88.1)	1420/1,566 (90.7)
Leak＜3mm	2/120 (1.7)	33/389 (8.5)	18/260 (6.9)	71/747 (9.5)	123/1,566 (7.9)
Leak 3-5 mm	2/120 (1.7)	6/389 (1.5)	8/260 (3.1)	17/747 (2.3)	22/1,566 (1.4)
Leak＞5 mm	0/120 (0.0)	1/389 (0.3)	0/260 (0.0)	1/747 (0.1)	1/1,566 (0.1)
**Anesthesia**					
General anesthesia	78/122 (63.9)	209/390 (53.6)	212/261 (81.2)	498/752 (66.2)	812/1,571 (51.7)
Localized anesthesia	44/122 (36.1)	181/390 (46.4)	49/261 (18.8)	254/752 (33.8)	759/759 (48.3)
**Imaging guidance**					
Fluoroscopy	0/122 (0.0)	32/390 (8.2)	14/261 (5.4)	102/752 (13.6)	345/1,571 (22.0)
TEE	115/122 (94.3)	319/390 (81.8)	245/261 (93.9)	636/752 (84.6)	1,193/1,571 (75.9)
ICE	7/122 (5.7)	39/390 (10.0)	2/261 (0.8)	14/752 (1.9)	33/1,571 (2.1)
Combined Radiofrequency Ablation/Cryoablation	76/122 (63.3)	200 /390 (51.3)	42/261 (16.1)	247/752 (32.9)	732/839 (46.6)

© 2022 Su F et al. *JAMA Network Open.*

eTable 8. Subgroups Analyses by the Composite End Point of Death, Stroke, and Systemic Embolism

Death, stroke or embolism at 0-7 days

Subgroup	Events rate	95%CI	P
**Age**			0.135
<75y	0.18 (4/2,182)	0.00 −0.36	
≥75y	0.55 (5/914)	0.07 −1.03	
**Sex**			0.182
Male	0.17 (3/1,782)	0.00 −0.36	
Famale	0.46 (6/1,314)	0.09 −0.82	
**BMI**			0.740
BMI<25	0.07 (6/1,764)	0.07 −0.61	
BMI≥25	0.23 (3/1,332)	0.00 −0.48	
**Smoking**			0.698
Current smoker	0.16 (1/619)	0.00 −0.48	
No smoking	0.32 (8/2,477)	0.10 −0.55	
**Previous stroke**			0.316
Yes	0.42 (6/1,418)	0.09 −0.76	
No	0.18 (3/1,678)	0.00 −0.38	
**Diabetes**			0.226
Yes	0.56 (4/720)	0.01 −1.10	
No	0.21 (5/2,376)	0.03 −0.39	
**Hypertension**			0.289
Yes	0.38 (8/2,130)	0.12 −0.64	
No	0.10 (1/966)	0.00 −0.31	
**Coronary artery disease**			0.720
Yes	0.34 (3/879)	0.00 −0.73	
No	0.27 (6/2,217)	0.05 −0.49	
**Vascular disease**			**0.005**
Yes	0.54 (9/1,682)	0.19 −0.88	
No	0.00 (0/1,414)		
**Chronic heart failure**			>0.999
Yes	0.00 (0/177)		
No	0.31 (9/2,919)	0.11 −0.51	
**LVEF %**			>0.999
>40	0.30 (9/2,990)	0.10 −0.50	
≤40	0.00 (0/106)		
**Bleeding history or predisposition**			0.055
Yes	0.96 (3/314)	0.00 −2.03	
No	0.22 (6/2,782)	0.04 −0.39	
**CHA2DS2−VASc score**			**0.032**
≥5	0.59 (7/1,188)	0.15 −1.02	
<5	0.10 (2/1,908)	0.00 −0.25	
**HAS−BLED score**			0.088
≥3	0.50 (7/1,397)	0.13 −0.87	
<3	0.12 (2/1,699)	0.00 −0.28	

0 0.5 1 1.5 2
Event rates 95%CI

Death, stroke or embolism at 8-30 days

Subgroup	Event rate (n/N)	95%CI	P
**Age**			0.206
<75y	0.14 (3/2,180)	0.00 −0.29	
≥75y	0.44 (4/914)	0.01 −0.87	
**Sex**			>0.999
Male	0.22 (4/1,781)	0.00 −0.44	
Famale	0.23 (3/1,313)	0.00 −0.49	
**BMI**			0.251
BMI<25	0.34 (6/1,762)	0.07 −0.61	
BMI≥25	0.08 (1/1,332)	0.00 −0.22	
**Smoking**			>0.999
Current smoker	0.16 (1/618)	0.00 −0.48	
No smoking	0.24 (6/2,476)	0.05 −0.44	
**Previous stroke**			>0.999
Yes	0.21 (3/1,416)	0.00 −0.45	
No	0.24 (4/1,678)	0.01 −0.47	
**Diabetes**			>0.999
Yes	0.14 (1/719)	0.00−0.41	
No	0.25 (6/2,375)	0.05 −0.45	
**Hypertension**			>0.999
Yes	0.23 (5/2,128)	0.03 −0.44	
No	0.21 (2/966)	0.00 −0.49	
**Coronary artery disease**			0.202
Yes	0.00 (0/878)		
No	0.32 (7/2,216)	0.08 −0.55	
**Vascular disease**			0.134
Yes	0.36 (6/1,680)	0.07−0.64	
No	0.07 (1/1,414)	0.00 −0.21	
**Chronic heart failure**			**0.005**
Yes	1.69 (3/177)	0.00 −3.6	
No	0.14 (4/2,917)	0.00 −0.27	
**LVEF %**			0.217
>40	0.20 (6/2,988)	0.04 −0.36	
≤40	0.94 (1/106)	0.00 −2.78	
**Bleeding history or predisposition**			0.153
Yes	0.64 (2/314)	0.00 −1.52	
No	0.18 (5/2,780)	0.02 −0.34	
**CHA2DS2−VASc score**			0.439
≥5	0.34 (4/1,186)	0.01 −0.67	
<5	0.16 (3/1,908)	0.00 −0.34	
**HAS−BLED score**			0.255
≥3	0.36 (5/1,395)	0.04 −0.67	
<3	0.12 (2/1,699)	0.00−0.28	

0 1 2 3
Event rates 95%CI

Death, stroke or embolism at 0-30 days

Subgroup	Event rate (n/N)	95%CI	P
**Age**			0.027
<75y	0.32 (7/2,182)	0.12 −0.56	
≥75y	0.98 (9/914)	0.34 −1.62	
**Sex**			0.314
Male	0.39 (7/1,782)	0.10 −0.68	
Famale	0.68 (9/1,314)	0.24 −1.13	
**BMI**			0.206
BMI<25	0.68 (12/1,764)	0.30 −1.06	
BMI≥25	0.30 (4/1,332)	0.01 −0.59	
**Smoking**			0.753
Current smoker	0.32 (2/619)	0.00 −0.77	
No smoking	0.57 (14/2,477)	0.27 −0.86	
**Previous stroke**			0.456
Yes	0.63 (9/1,418)	0.22 −1.05	
No	0.42 (7/1,678)	0.11 −0.73	
**Diabetes**			0.551
Yes	0.69 (5/720)	0.09 −1.30	
No	0.46 (11/2,376)	0.19 −0.74	
**Hypertension**			0.418
Yes	0.61 (13/2,130)	0.28 −0.94	
No	0.31 (3/966)	0.00 −0.66	
**Coronary artery disease**			0.580
Yes	0.34 (3/879)	0.07 −0.99	
No	0.59 (13/2,217)	0.27 −0.90	
**Vascular disease**			**0.002**
Yes	0.89 (15/1,682)	0.44 −1.34	
No	0.07 (1/1,414)	0.00 −0.21	
**Chronic heart failure**			0.059
Yes	1.69 (3/177)	0.35 −4.87	
No	0.45 (13/2,919)	0.20 −0.69	
**LVEF %**			0.428
>40	0.50 (15/2,990)	0.25 −0.75	
≤40	0.94 (1/106)	0.00−2.78	
**Bleeding history or predisposition**			**0.018**
Yes	1.59 (5/314)	0.21 −2.98	
No	0.40 (11/2,782)	0.16 −0.63	
**CHA2DS2−VASc score**			**0.018**
≥5	0.93 (11/1,188)	0.38 −1.47	
<5	0.26 (5/1,908)	0.03 −0.49	
**HAS−BLED score**			**0.021**
≥3	0.86 (12/1,397)	0.38 −1.34	
<3	0.24 (4/1,699)	0.00 −0.47	

0 1 2 3 4 5
Event rates 95%CI

© 2022 Su F et al. *JAMA Network Open.*

eTable 9. Subgroups Analyses by the Composite End Point of Any Life-threatening or Major Bleeding Events

Any life-threatening or major bleeding at 0-7 days

Subgroups	Events rate (n/N)	95%CI	P
**Age**			**0.041**
<75y	0.87 (19/2,182)	0.45−1.26	
≥75y	1.75 (16/914)	0.90−2.60	
**Sex**			0.305
Male	0.95 (17/1,782)	0.50−1.41	
Famale	1.37 (18/1,314)	0.74−2.00	
**BMI**			0.735
BMI<25	1.08 (19/1,764)	0.60−1.56	
BMI≥25	1.20 (16/1,332)	0.62−1.79	
**Smoking**			0.833
Current smoker	0.97 (6/619)	0.20−1.74	
No smoking	1.17 (29/2,477)	0.75−1.59	
**Previous stroke**			0.294
Yes	1.34 (19/1,418)	0.74−1.94	
no	0.95 (16/1,678)	0.49−1.42	
**Diabetes**			0.312
Yes	0.69 (5/720)	0.09−1.30	
no	1.26 (30/2,376)	0.81−1.71	
**Hypertension**			>0.999
Yes	1.13 (24/2,130)	0.68−1.58	
no	1.14 (11/966)	0.47−1.81	
**Coronary artery disease**			0.061
Yes	1.71 (15/879)	0.85−2.56	
no	0.90 (20/2,217)	0.51−1.30	
**Vascular disease**			0.123
Yes	1.43 (24/1,682)	0.86−1.99	
no	0.78 (11/1,414)	0.32−1.24	
**Chronic heart failure**			0.262
Yes	0.00 (0/177)		
no	1.20 (35/2,919)	0.80−1.59	
**LVEF %**			>0.999
>40	1.14 (34/2,990)	0.76−1.52	
≤40	0.94 (1/106)	0.00−2.78	
**Bleeding history or predisposition**			0.081
Yes	2.23 (7/314)	0.60−3.86	
no	1.01 (28/2,782)	0.64−1.38	
**CHA2DS2−VASc score**			0.224
≥5	1.43 (17/1,188)	0.76−2.28	
<5	0.94 (18/1,908)	0.51−1.38	
**HAS−BLED score**			0.307
≥3	1.36 (19/1,397)	0.75−1.97	
<3	0.94 (16/1,699)	0.48−1.40	

0 1 2 3 4 5

Event Rate 95%CI

Any life-threatening or major bleeding at 8-30days

Subgroups	Event Rate (n/N)	95%CI	P
**Age**			0.560
<75y	0.14 (3/2,180)	0.00−0.29	
≥75y	0.00 (0/914)		
**Sex**			>0.999
Male	0.11 (2/1,781)	0.00−0.27	
Famale	0.08 (1/1,313)	0.00−0.23	
**BMI**			0.264
BMI<25	0.17 (3/1,762)	0.00−0.36	
BMI≥25	0.00 (0/1,332)		
**Smoking**			0.488
Current smoker	0.16 (1/618)	0.00−0.48	
No smoking	0.08 (2/2,476)	0.00−0.19	
**Previous stroke**			>0.999
Yes	0.07 (1/1,416)	0.00−0.21	
no	0.12 (2/1,678)	0.00−0.28	
**Diabetes**			>0.999
Yes	0.00 (0/719)		
no	0.13 (3/2,375)	0.00−0.27	
**Hypertension**			0.232
Yes	0.05 (1/2,128)	0.00−0.14	
no	0.21 (2/966)	0.00−0.49	
**Coronary artery disease**			0.563
Yes	0.00 (0/878)		
no	0.14 (3/2,216)	0.00−0.29	
**Vascular disease**			>0.999
Yes	0.12 (2/1,680)	0.00−0.28	
no	0.07(1/1,414)	0.00−0.21	
**Chronic heart failure**			**0.009**
Yes	1.13 (2/177)	0.00−2.69	
no	0.03 (1/2,917)	0.00−0.10	
**LVEF %**			>0.999
>40	0.10 (3/2,988)	0.00−0.21	
≤40	0.00 (0/106)		
**Bleeding history or predisposition**			>0.999
Yes	0.00 (0/314)		
no	0.11 (3/2,780)	0.00−0.23	
**CHA2DS2−VASc score**			0.291
≥5	0.00 (0/1,186)		
<5	0.16 (3/1,908)	0.00−0.34	
**HAS−BLED score**			>0.999
≥3	0.07 (1/1,395)	0.00−0.21	
<3	0.12 (2/1,699)	0.00−0.28	

0 1 2

Event Rate 95%CI

Any life-threatening or major bleeding at 0-30days

Subgroups	Events rate (n/N)	95%CI	P
**Age**			0.106
<75y	1.01 (22/2,182)	0.59−1.43	
≥75y	1.75 (16/914)	0.90−2.60	
**Sex**			0.409
Male	1.07 (19/1,782)	0.59−1.66	
Famale	1.45 (19/1,314)	0.80−2.09	
**BMI**			>0.999
BMI<25	1.25 (22/1,764)	0.73−1.77	
BMI≥25	1.20 (16/1,332)	0.62−1.79	
**Smoking**			>0.999
Current smoker	1.13 (7/619)	0.30−1.96	
No smoking	1.25 (31/2,479)	0.81−1.69	
**Previous stroke**			0.417
Yes	1.41 (20/1,418)	0.80−2.02	
no	1.07 (18/1,678)	0.58−1.57	
**Diabetes**			0.176
Yes	0.69 (5/720)	0.09−1.30	
no	1.39 (33/2,376)	0.92−1.86	
**Hypertension**			0.725
Yes	1.17 (25/2,130)	0.72−1.63	
no	1.35 (13/966)	0.62−2.07	
**Coronary artery disease**			0.147
Yes	1.71 (15/879)	0.85−2.56	
no	1.04 (23/2,217)	0.62−1.46	
**Vascular disease**			0.101
Yes	1.55 (26/1,682)	0.96−2.14	
no	0.85 (12/1,414)	0.37−1.33	
**Chronic heart failure**			>0.999
Yes	1.13 (2/177)	0.14−4.02	
no	1.23 (36/2,919)	0.83−1.63	
**LVEF %**			>0.999
>40	1.24 (37/2,990)	0.84−1.63	
≤40	0.94 (1/106)	0.00−5.14	
**Bleeding history or predisposition**			0.100
Yes	2.23 (7/314)	0.60−3.86	
no	1.11 (31/2,782)	0.72−1.50	
**CHA2DS2−VASc score**			0.408
≥5	1.43 (17/1,188)	0.76−2.11	
<5	1.10 (21/1,908)	0.68−1.68	
**HAS−BLED score**			0.413
≥3	1.43 (20/1,397)	0.81−2.05	
<3	1.06 (18/1,699)	0.57−1.55	

0 1 2 3 4 5

Event Rate 95%CI

© 2022 Su F et al. *JAMA Network Open.*

eTable 10. Subgroups Analyses by the Composite End Point of Death, Stroke, Systemic Embolism, and Any Life-threatening or Major Bleeding Events

Death, stroke, systemic embolism, and life threatening or major bleeding at 0-7 days

Subgroups	Events rate (n/N)	95%CI	P
**Age**			**0.039**
<75y	1.05 (23/2,182)	0.63−1.48	
≥75y	2.08 (19/914)	1.15−3.00	
**Sex**			0.210
Male	1.12 (20/1,782)	0.63−1.61	
Famale	1.67 (22/1,314)	0.98−2.37	
**BMI**			>0.999
BMI<25	1.36 (24/1,764)	0.82−1.90	
BMI≥25	1.35 (18/1,332)	0.73−1.97	
**Smoking**			0.700
Current smoker	1.13 (7/619)	0.30−1.96	
No smoking	1.41 (35/2,477)	0.95−1.88	
**Previous stroke**			0.276
Yes	1.62 (23/1,418)	0.96−2.28	
no	1.13 (19/1,678)	0.63−1.64	
**Diabetes**			0.586
Yes	1.11 (8/720)	0.35−1.88	
no	1.43 (34/2,376)	0.95−1.91	
**Hypertension**			0.867
Yes	1.41 (30/2,130)	0.91−2.00	
no	1.24 (12/966)	0.54−1.94	
**Coronary artery disease**			0.086
Yes	1.93 (17/879)	1.02−2.84	
no	1.13 (25/2,217)	0.69−1.57	
**Vascular disease**			**0.012**
Yes	1.84 (31/1,682)	1.20−2.49	
no	0.78 (11/1,414)	0.32−1.24	
**Chronic heart failure**			0.172
Yes	0.00 (0/177)	–	
no	1.44 (42/2,919)	1.01−1.87	
**LVEF %**			>0.999
>40	1.37 (41/2,990)	0.95−1.79	
≤40	0.94 (1/106)	0.00−2.78	
**Bleeding history or predisposition**			0.067
Yes	2.55 (8/314)	0.80−4.29	
no	1.22 (34/2,782)	0.81−1.63	
**CHA2DS2−VASc score**			0.078
≥5	1.85 (22/1,188)	1.08−2.62	
<5	1.05 (20/1,908)	0.59 −1.51	
**HAS−BLED score**			0.121
≥3	1.72 (24/1,400)	1.04−2.40	
<3	1.06 (18/1,699)	0.57−1.55	

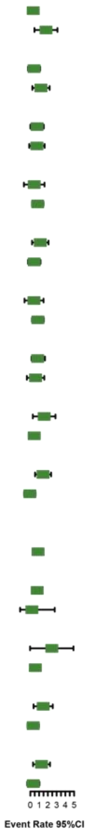

Death, stroke, systemic embolism, and life threatening or major bleeding at 8-30 days

Subgroups	Events rate (n/N)	95%CI	P
**Age**			0.246
<75y	0.18 (4/2,180)	0.00−0.36	
≥75y	0.44 (4/914)	0.01−0.87	
**Sex**			>0.999
Male	0.28 (5/1,781)	0.09−0.65	
Famale	0.23 (3/1,313)	0.00−0.49	
**BMI**			0.149
BMI<25	0.40 (7/1,762)	0.10−0.69	
BMI≥25	0.08 (1/1,332)	0.00−0.22	
**Smoking**			0.664
Current smoker	0.32 (2/618)	0.00−0.77	
No smoking	0.24 (6/2,476)	0.05−0.44	
**Previous stroke**			>0.999
Yes	0.28 (4/1,416)	0.01−0.56	
no	0.24 (4/1,678)	0.01−0.47	
**Diabetes**			0.690
Yes	0.14 (1/719)	0.00−0.41	
no	0.29 (7/2,375)	0.08−0.51	
**Hypertension**			0.711
Yes	0.23 (5/2,128)	0.03−0.44	
no	0.31 (3/966)	0.00−0.66	
**Coronary artery disease**			0.115
Yes	0.00 (0/878)		
no	0.36 (8/2,216)	0.11−0.61	
**Vascular disease**			0.303
Yes	0.36 (6/1,680)	0.07−0.64	
no	0.14 (2/1,414)	0.00−0.34	
**Chronic heart failure**			**0.008**
Yes	1.69 (3/177)	0.00−3.60	
no	0.17 (5/2,917)	0.02−0.32	
**LVEF %**			0.244
>40	0.23 (7/2,988)	0.06−0.41	
≤40	0.94 (1/106)	0.00−2.78	
**Bleeding history or predisposition**			0.191
Yes	0.64 (2/314)	0.00−1.52	
no	0.22 (6/2,780)	0.04−0.39	
**CHA2DS2−VASc score**			0.492
≥5	0.34 (4/1,186)	0.01−0.67	
<5	0.21 (4/1,908)	0.00 −0.41	
**HAS−BLED score**			0.480
≥3	0.36 (5/1,395)	0.04−0.67	
<3	0.18 (3/1,699)	0.00−0.38	

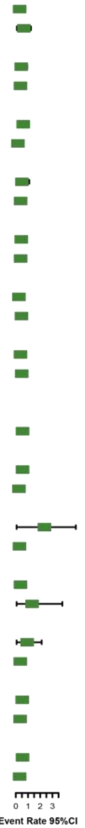

Death, stroke, systemic embolism, and life threatening or major bleeding at 0-30 days

Subgroups	Events rate (n/N)	95%CI	P
**Age**			**0.012**
<75y	1.24 (27/2,182)	0.77−1.70	
≥75y	2.52 (23/914)	1.50−3.53	
**Sex**			0.313
Male	1.40 (25/1,782)	0.86−1.95	
Famale	1.90 (25/1,314)	1.16−2.64	
**BMI**			0.565
BMI<25	1.76 (31/1,764)	1.14−2.37	
BMI≥25	1.43 (19/1,332)	0.79−2.06	
**Smoking**			0.859
Current smoker	1.45 (9/619)	0.51−2.40	
No smoking	1.66 (41/2,477)	1.15−2.16	
**Previous stroke**			0.255
Yes	1.90 (27/1,418)	1.19−2.62	
no	1.37 (23/1,678)	0.81−1.93	
**Diabetes**			0.499
Yes	1.25 (9/720)	0.44−2.06	
no	1.73 (41/2,376)	1.20−2.25	
**Hypertension**			>0.999
Yes	1.64 (35/2,130)	1.10−2.18	
no	1.55 (15/966)	0.77−2.33	
**Coronary artery disease**			0.429
Yes	1.93 (17/879)	1.02−2.84	
no	1.49 (33/2,217)	0.98−1.99	
**Vascular disease**			**0.006**
Yes	2.20 (37/1,682)	1.50−2.90	
no	0.92 (13/1,414)	0.42−1.42	
**Chronic heart failure**			0.762
Yes	1.69 (3/177)	0.35−4.87	
no	1.61 (47/2,919)	1.15−2.07	
**LVEF %**			0.689
>40	1.61 (48/2,990)	1.15−2.06	
≤40	1.89 (2/1,060)	0.00−4.48	
**Bleeding history or predisposition**			**0.030**
Yes	3.18 (10/314)	1.24−5.13	
no	1.44 (40/2,782)	1.00−1.88	
**CHA2DS2−VASc score**			0.056
≥5	2.19 (26/1,188)	1.36−3.02	
<5	1.26 (24/1,908)	0.76 −1.76	
**HAS−BLED score**			0.085
≥3	2.08 (29/1,397)	1.33−2.82	
<3	1.24 (21/1,699)	0.71−1.76	

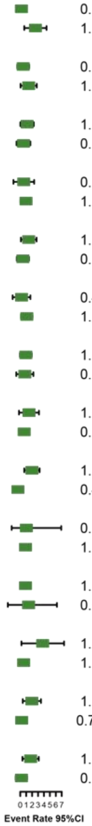

© 2022 Su F et al. *JAMA Network Open.*

eTable 11. Associations of Procedural Configurations With Outcomes

30-day events	Events/N (%)	Univariable		Multivariate Model 1		Multivariate Model 2	
		OR (95%CI)	P	OR (95%CI)	P	OR (95%CI)	P
Procedural success							
TEE	2,452/2,508 (97.8)	Ref		Ref		Ref	
ICE	93/95 (97.9)	1.06 (0.26, 4.42)	0.739	1.35 (0.30, 6.04)	0.992	1.53 (0.34, 6.88)	0.852
Fluoroscopy	487/493 (98.8)	1.85 (0.79, 4.34)	0.285	1.81 (0.70, 4.68)	0.441	1.75 (0.67, 4.56)	0.543
Death, stroke, systemic embolism							
TEE	14/2,508 (0.6)	Ref		Ref		Ref	
ICE	0/95 (0.0)	-	0.975	-	0.972	-	0.972
Fluoroscopy	2/493 (0.4)	0.73 (0.16, 3.20)	0.976	0.68 (0.12, 3.85)	0.974	0.67 (0.11, 3.93)	0.974
Any life-threatening or major bleeding							
TEE	33/2,508 (1.3)	Ref		Ref		Ref	
ICE	2/95 (2.1)	1.61 (0.38, 6.82)	0.264	1.08 (0.23, 5.05)	0.568	0.90 (0.19, 4.34)	0.710
Fluoroscopy	3/493 (0.6)	0.46 (0.14, 1.50)	0.138	0.47 (0.13, 1.71)	0.261	0.44 (0.12, 1.64)	0.292

© 2022 Su F et al. *JAMA Network Open.*

eTable 12. Baseline Characteristics Patients Who Underwent of Noncombined vs Combined Radiofrequency Ablation and Cryoablation

Characteristics	Non-radiofrequency Ablation/Cryoablation (N=1799)	Combined-Radiofrequency Ablation/Cryoablation (N=1297)
Age, yrs	69.9±9.5	68.1±9.1
65-74 years	718 (39.9)	595 (45.9)
≥75y	607 (33.7)	307 (23.7)
Male	1015 (56.4)	767 (59.1)
Body mass index (BMI)	24.63±3.55	25.08±3.52
Heart rate, per/min	81.28±19.36	82.92±21.95
Diabetes	400 (23.5)	320 (24.7)
Previous Stroke	855 (47.5)	563 (43.4)
Ischemic stroke or TIA	830 (46.1)	550 (42.4)
Hemorrhagic stroke	71 (3.9)	36 (2.8)
Hypertension	1255 (69.8)	875 (67.5)
Coronary artery disease	533 (29.6)	346 (26.7)
Previous PCI	209 (11.6)	118 (9.1)
Previous CABG	26 (1.4)	20 (1.5)
Vascular disease	962 (53.5)	719 (55.4)
Current Smoker	181 (10.1)	153 (11.8)
Alcohol abuse	93 (5.2)	76 (5.9)
Chronic heart failure	270 (15.0)	192 (14.8)
LVEF (%)	59.45±8.43	60.86±8.11
Abnormal thyroidal function	81 (4.5)	52 (4.0)
Abnormal renal function	46 (2.6)	26 (2.0)
Abnormal liver function	31 (1.7)	20 (1.5)
Bleeding history or predisposition	212 (11.8)	102 (7.9)
Concomitant use of drugs	612 (34.0)	429 (33.1)
Classification of AF		
Paroxysmal	613 (34.1)	636 (49.0)
Persistent	798 (44.4)	479 (36.9)
Long-standing persistent (>1y)/ Permanent	388 (21.5)	182 (14.0)
CHA_2DS_2-VASc score	4.07±1.82	3.84±1.77
HAS-BLED score	2.47±1.18	2.30±1.12
ATRIA score	6.41±2.93	5.95±2.95
Recaptured (≥2 times) before release	140 (7.9)	118 (9.1)
Device used	1,865 (1.04 per patient)	1340/1297 (1.03 per patient)

© 2022 Su F et al. *JAMA Network Open.*

Kissing-devices	3 (0.2)	2 (0.2)
Device size		
21 mm	96/1791	92/1291
24 mm	344/1791	290/1291
27 mm	521/1791	397/1291
30 mm	436/1791	278/1291
33 mm	397/1791	236/1291
Residual peri-device leakage		
Complete sealing	1576 (88.0)	1194 (92.5)
Leak < 3mm	171 (9.5)	78 (6.0)
Leak 3-5 mm	41 (2.3)	19 (1.5)
Leak > 5 mm	3 (0.2)	0 (0.0)
Anesthesia		
General anesthesia	606 (33.8)	677 (52.8)
Localized anesthesia	1185 (66.2)	614 (34.1)
Imaging guidance		
TEE	1565 (87.0)	943 (72.7)
Fluoroscopy	189 (10.5)	304 (23.4)
ICE	45 (2.5)	50 (3.9)

© 2022 Su F et al. *JAMA Network Open.*

eTable 13. Clinical Events of Patients Who Underwent of Noncombined vs Combined Radiofrequency Ablation and Cryoablation at 30 Days After the Procedure

	Non radiofrequency Ablation/Cryoablation (N=1799)				Combined Radiofrequency Ablation/Cryoablation (N=1297)			
	≤7d	8-30 days	Total		≤7d	8-30 days	Total	
	Events	Events	Events	Event rate (95%CI)	Events	Events	Events	Event rate (95%CI)
Device success	-	-	1,799	99.6 (99.1, 99.8)	-	-	1,291	99.5 (99.0, 99.8)
Technical success	-	-	1,781	99.0 (98.4, 99.4)	-	-	1,287	99.2 (98.6, 99.6)
Procedure success	-	-	1,763	98.0 (97.2, 98.6)	-	-	1,269	97.8 (97.1, 98.6)
Death, stroke, systemic embolism	3	5	8	0.44 (0.19, 0.87)	6	2	8	0.62 (0.27, 1.21)
Death, stroke, systemic embolism, and any life-threatening or major bleeding	24	4	28	1.56 (1.04, 2.24)	16	6	22	1.70 (1.07, 2.56)
Individual components								
Death	0	4	4	0.22 (0.06, 0.57)	2	2	4	0.31 (0.08, 0.79)
-Cardiovascular death	0	3	3	0.17 (0.03, 0.49)	2	2	4	0.31 (0.08, 0.79)
Pulmonary embolism	0	1	1	-	0	0	0	-
Cardiac arrest	0	0	0	-	2	1	3	-
Hemorrhagic stroke	0	1	1	-	0	0	0	-
Gastrointestinal bleeding	0	0	0	-	0	1	1	-
Unknown cause	0	1	1	-	0	0	0	-
-Non-cardiovascular death	0	1	1	-	0	0	0	-
Pneumonia	0	1	1	-	0	0	0	-

© 2022 Su F et al. *JAMA Network Open.*

Stroke	3	2	5	0.28 (0.09, 0.65)	4	0	4	0.31 (0.08, 0.79)
-Hemorrhagic stroke	1	1	2	0.11 (0.01, 0.40)	1	0	1	0.08 (0.00, 0.43)
-Ischemic stroke	2	1	3	0.17 (0.03, 0.49)	3	0	3	0.23 (0.05, 0.67)
TIA	1	1	2	0.11 (0.01, 0.40)	0	0	0	0.00 (0.00, 0.28)
Systemic embolism	0	1	1	0.06 (0.00,0.31)	0	0	0	0.00 (0.00, 0.28)
Procedural complications	22	0	22	1.22 (0.77, 1.85)	15	0	15	1.16 (0.65, 1.90)
-Vascular access-related complications	2	0	2	0.11 (0.01, 0.40)	1	0	1	0.08 (0.00, 0.43)
-Device-related complications	8	0	8	0.44 (0.19, 0.87)	4	0	4	0.31 (0.08, 0.79)
Cardiac tamponade	8	0	8	0.44 (0.19, 0.87)	3	0	3	0.23 (0.05, 0.67)
Pneumothorax	1	0	1	0.06 (0.00,0.31)	0	0	0	0.00 (0.00, 0.28)
-Pericardial effusion	10	0	10	0.56 (0.27, 1.02)	10	0	10	0.77 (0.37, 1.41)
-Others	2	0	2	0.11 (0.01, 0.40)	0	0	0	0.00 (0.00, 0.28)
Esophageal	1	0	1	0.06 (0.00,0.31)	0	0	0	0.00 (0.00, 0.28)
Adverse reaction to anesthesia	1	0	1	0.06 (0.00,0.31)	0	0	0	0.00 (0.00, 0.28)
Any Bleeding	23	7	30	1.67 (1.13, 2.37)	16	6	22	1.70 (1.07, 2.56)
LAAO Munich consensus classification								
-Any Life-threatening/major bleeding	20	2	22	1.22 (0.77, 1.85)	15	2	16	1.23 (0.71, 2.00)
-Life threatening or disabling	1	2	3	0.17 (0.03, 0.49)	1	1	2	0.16 (0.02, 0.61)
-Major bleeding	20	0	20	1.11 (0.68, 1.71)	13	0	13	1.00 (0.53, 1.71)
-Minor bleeding	2	6	8	0.44 (0.19, 0.87)	1	5	6	0.46 (0.17, 1.00)
BARC classification								

© 2022 Su F et al. *JAMA Network Open.*

- Type 5	0	1	1	0.06 (0.00,0.31)	0	1	1	0.08 (0.00, 0.43)
- Type 3	10	1	11	0.61 (0.31, 1.09)	5	0	5	0.39 (0.13, 0.90)
Type 3c	1	1	2	0.11 (0.01, 0.40)	1	0	1	0.08 (0.00, 0.43)
Type 3b	9	0	9	0.50 (0.23, 0.95)	4	0	4	0.31 (0.08, 0.79)
-Type 2	13	5	18	1.00 (0.59, 1.58)	11	5	16	1.23 (0.71, 2.00)

© 2022 Su F et al. *JAMA Network Open.*

eTable 14. LAAO Registries Conducted in Asia

LAAO registries conducted in Asia					
Study	WASP (Asian cohort)	Korean Registry	SALUTE (Japan)	Fuwai Registry	RECORD
Number of pts	201	96	42	658	3,096
Age, yrs	70.8	65.1	72.5	67.7	69.1
CHASD2VASc score	4.1	4.1	3.6	3.7	4.0
HAS-BLED score	2.2	2.8	2.9	2.5	2.4

© 2022 Su F et al. *JAMA Network Open.*

eTable 15. Clinical Events at 45 Days After LAAO

	≤7d	8-45 days	Total	
	**Events**	**Events**	**Events**	**Event rate (95%CI)**
Device success	-	-	3,082	99.5 (99.3-99.8)
Technical success	-	-	3,068	99.1 (98.8-99.4)
Procedure success	-	-	3,032	97.9 (97.4-98.4)
Death, stroke, systemic embolism	9	27	36	1.16 (0.82-1.61)
Death, stroke, systemic embolism, and any life-threatening or major bleeding	40	38	78	2.52 (2.00-3.13)
Individual components				
Death	2	14	16	0.52 (0.30-0.84)
-Cardiovascular death	2	13	15	0.48 (0.27-0.80)
-Non-cardiovascular death	0	1	1	0.03 (0.00-0.18)
Stroke	7	12	19	0.61 (0.37-0.96)
-Hemorrhagic stroke	2	4	6	0.19 (0.07-0.42)
-Ischemic stroke	5	8	13	0.42 (0.22-0.72)
TIA	1	3	4	0.13 (0.04-0.33)
Systemic embolism	0	1	1	0.03 (0.00-0.18)
Procedural complications	37	0	37	1.20 (0.84-1.64)
Any Bleeding	39	25	63	2.03 (1.57-2.60)
LAAO Munich consensus classification				
-Any Life-threatening/major bleeding	35	13	48	1.55 (1.15-2.05)
-Life threatening or disabling	2	6	8	0.26 (0.11-0.51)
-Major bleeding	33	7	40	1.36 (0.98-1.83)
-Minor bleeding	3	12	15	0.48 (0.27-0.80)
BARC classification				
- Type 5	0	4	4	0.13 (0.04-0.33)
- Type 3	15	5	20	0.65 (0.40-1.00)
- Type 2	24	15	39	1.26 (0.90-1.72)
